# Causal association between hypothyroidism and obstructive sleep apnea: A bidirectional 2-sample Mendelian Randomization study

**DOI:** 10.1097/MD.0000000000040114

**Published:** 2024-10-18

**Authors:** Ning Lu, Bi Chen, Pingli Liu, Cuocuo Wang, Zhaojun Lu, Shengli Li

**Affiliations:** a Department of General Practice, Affiliated Hospital of Xuzhou Medical University, Xuzhou, China; b Department of Pulmonary and Critical Care Medicine, Affiliated Hospital of Xuzhou Medical University, Xuzhou, China; c School of Public Health, Xuzhou Medical University, Xuzhou, China; d Department of Clinical Research Institute, Affiliated Hospital of Xuzhou Medical University, Xuzhou, China.

**Keywords:** hypothyroidism, Mendelian randomization, obstructive sleep apnea

## Abstract

Although previous epidemiological studies have investigated the correlation between hypothyroidism and obstructive sleep apnea (OSA), the results are controversial and conflicting. Therefore, we used a bidirectional 2-sample Mendelian randomization (MR) approach to infer the causal relationship between hypothyroidism and OSA. We performed a bidirectional 2-sample MR analysis to infer the causal relationship between hypothyroidism and OSA using genome-wide association study (GWAS) data. The hypothyroidism dataset was obtained from GWAS of the IEU database (https://gwas.mrcieu.ac.uk/). The GWAS dataset associated with OSA was obtained from the FinnGen Biobank (https://www.finngen.fi/en). MR results were estimated using the inverse variance weighted, weighted median, MR-Egger, simple mode, and weighted mode methods. Sensitivity analysis was conducted using the heterogeneity, pleiotropy, and leave-one-out tests. Scatter plots, forest plots, funnel plots, and leave-one-out plots were used as visualizations of MR results. According to the inverse variance weighted method, forward MR analysis showed that hypothyroidism was significantly associated with OSA (odds ratio, 1.870 [95% confidence interval, 1.055–3.315]; *P* = .032). There was no evidence to suggest a causal relationship between OSA and the risk of hypothyroidism in reverse MR analysis (*P* = .881). Furthermore, sensitivity analysis further confirmed the robust results. Our bidirectional 2-sample MR analysis revealed that hypothyroidism could increase the risk of developing OSA but did not provide evidence to support a causal relationship of OSA on hypothyroidism. Thus, patients with hypothyroidism should strengthen their sleep quality monitoring, and further research is needed to understand the role of hypothyroidism effects on OSA.

## 1. Introduction

Hypothyroidism, a common endocrine disorder characterized by thyroid hormone deficiency, affects millions of individuals worldwide.^[1,2]^ Thyroid hormones affect many bodily systems and play a crucial role in regulating metabolism, growth, neuronal development, and energy expenditure.^[3,4]^ If left untreated, hypothyroidism can have several negative consequences. Emerging evidence suggests potential associations between hypothyroidism and various comorbidities, including cardiovascular diseases, dyslipidemia, depression, and sleep disorders.^[5–10]^ Of particular interest is the proposed link between hypothyroidism and sleep apnea (SA).

SA is a potentially serious sleep disorder characterized by recurrent interruptions in breathing during sleep.^[11]^ More than 1 billion individuals worldwide currently experience some form of SA.^[12]^ Obstructive sleep apnea (OSA) is the main type of SA, and it has emerged as a serious public health problem because of its high prevalence and adverse consequences.^[13]^

Previous studies are prone to many biases inherent in observational designs, such as confounding and reverse causality biases.^[14,15]^ Randomized controlled trials (RCTs) are considered the gold standard for making causal inferences in health sciences.^[15]^ However, RCTs need a large amount of financial and material resources and are also impractical in some cases. Mendelian randomization (MR), a method that uses genetic variants as instrumental variables (IVs) to evaluate the causal effect of exposure on outcome, has been widely used to infer causal relationships.^[15,16]^ The process of MR is similar to a natural RCT, which can overcome the shortcomings of traditional observational epidemiological studies.^[17]^

In this study, we applied a bidirectional 2-sample MR design to explore the causal relationship between hypothyroidism and OSA and to provide new strategies for clinical treatment.

## 2. Materials and methods

### 2.1. Study design

**Figure 1.**
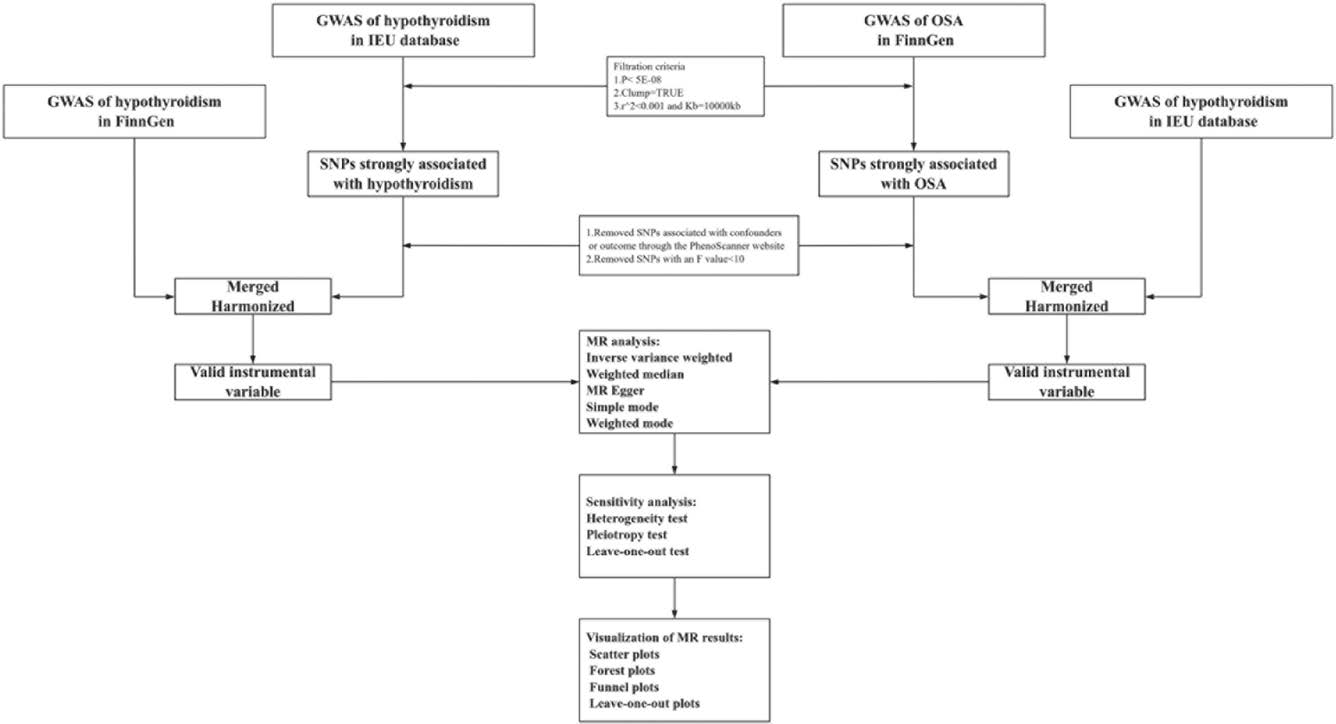
Flowchart of the bidirectional 2-sample Mendelian randomization study. GWAS = genome-wide association study, MR = Mendelian randomization, OSA = obstructive sleep apnea.

**Figure 2.**
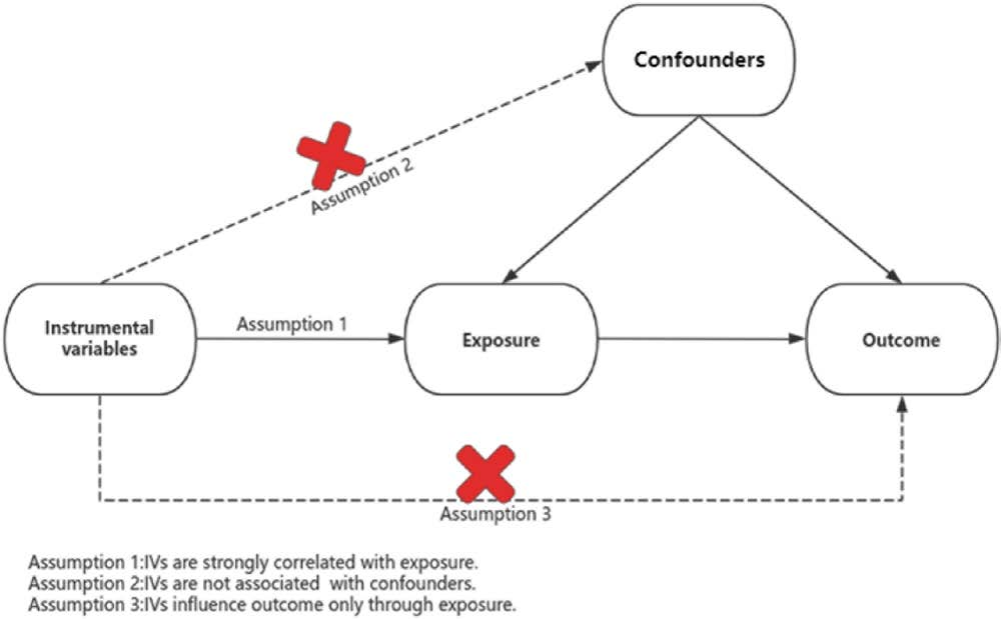
Three assumptions of Mendelian randomization model. IV = instrumental variable.

We conducted a bidirectional 2-sample MR analysis to assess the potential causal associations between hypothyroidism and OSA, followed by sensitivity analysis and visualization of the MR results. Our study was reported on the basis of the STROBE-MR statement.^[18]^ The processes of our study are presented in Figure 1. In MR analysis, IVs must satisfy 3 assumptions (Fig. 2): IVs are strongly associated with exposure (the relevance assumption), IVs are not associated with any potential confounding between exposure and outcome (the independence assumption), and IVs affect outcome only through exposure (the exclusion restriction assumption).^[16,19]^

Our analyses were performed using TwoSampleMR (version 0.6.2) and MR-PRESSO (1.0) packages in R (version 4.4.0).

### 2.2. Genome-wide association study summary data sources

We selected exposure and outcome genome-wide association study (GWAS) datasets from different databases, and all GWAS were restricted to individuals of European ancestry to avoid potential bias caused by population stratification.

The summary datasets of hypothyroidism were obtained from the IEU Open GWAS database involving a total of 473,703 samples (https://gwas.mrcieu.ac.uk/), with the ID number “ebi-a-GCST90029022.” A summary dataset of OSA was obtained from the FinnGen Biobank. FinnGen study is a large-scale genomics initiative that has analyzed over 500,000 Finnish biobank samples and correlated genetic variation with health data to understand disease mechanisms and predispositions.^[20]^ In this GWAS, OSA was diagnosed based on the International Statistical Classification of Diseases codes (ICD-10: G47.3; ICD-9: 3472A). Details of the GWAS data sources are presented in Table 1.

**Table 1:** Hypothyroidism and OSA GWAS datasets.

	Hypothyroidism	OSA
Year	2018	2021
Population	European	European
Sex	Males and Females	Males and Females
Sample size	473,703	217,955
GWAS ID	ebi-a-GCST90029022	finn-b-G6_SLEEPAPNO

GWAS = genome-wide association study, OSA = obstructive sleep apnea.

### 2.3. Selection of IVs

In the forward MR analysis, hypothyroidism was taken as exposure, and OSA was taken as an outcome. First, we identified single nucleotide polymorphisms (SNPs) related to hypothyroidism risk at *P* < 5 × 10^−8^, setting linkage disequilibrium $r^{2}<0.001$ within a 10,000-kb distance from the exposure GWAS dataset. Second, we removed SNPs associated with confounders and OSA through the PhenoScanner website (http://www.phenoscanner.medschl.cam.ac.uk/).^[21]^ Well-known risk factors for OSA included, but were not limited to obesity, advanced age, smoking, asthma, diabetes, hypertension, and alcohol consumption.^[12,22]^ To obtain strong IVs, the SNPs with an F value <10 were deleted. SNPs were then merged and harmonized for exposure and outcome, and palindromic or incompatible alleles were removed. Finally, the remaining SNPs were used as valid IVs for MR estimates, sensitivity analysis, and visualization of MR results.

We proceeded to identify the causality between OSA and hypothyroidism in the reverse direction, employing OSA as exposure and hypothyroidism as outcome. The procedure for selecting IVs was the same as that for the forward MR analysis described above. Risk factors for developing hypothyroidism included but were not limited to iodine deficiency, iodine excess, other autoimmune conditions, selenium deficiency, smoking, ethnicity, immunosuppressants, and endocrine disruptors.^[3,23]^ Details of the valid IVs are listed in Tables S1 and S2, http://links.lww.com/MD/N740.

### 2.4. Statistical analysis

Five methods were applied to estimate the causal relationship between hypothyroidism and OSA, including the inverse variance weighted (IVW), weighted median, MR-Egger, simple mode, and weighted mode methods. The IVW was selected as the primary statistical method. This method assumes that all SNPs are valid IVs and provides the highest precise causal estimates.^[24,25]^ The WM method can provide valid estimates when at least half of the SNPs satisfy the IV assumptions.^[26]^ Other complementary methods are used to evaluate the robustness of the results.

The F-statistic was employed to alleviate bias from weak instruments, and F < 10 means that the IV causes weak instrumental bias.^[26]^ Currently, there are 2 common formulas for calculating F statistics: $F=\left(\frac{n-k-1}{k}\right)\left(\frac{r^{2}}{1-r^{2}}\right)$ and $F=\frac{b^{2}}{se^{2}}$.^[23,27]^ We used $F=\frac{b^{2}}{se^{2}}$ (b, effect size [exposure]; se, standard error [exposure]) in our study.

### 2.5. Sensitivity analysis

Cochran Q of IVW was used to evaluate the heterogeneity in valid IVs.^[28]^ The Mendelian randomization pleiotropy residual sum and outlier (MR-PRESSO) approach was applied to detect and correct for horizontal pleiotropy, which identified horizontal pleiotropic outliers and generated new estimates by removing pleiotropic outliers.^[29]^ The intercept of MR-Egger was also employed to evaluate the horizontal pleiotropy of the selected IVs.^[30,31]^ Furthermore, the leave-one-out sensitivity test was used to assess the robustness of the results by examining whether the MR results were driven after individually removing a valid IV.^[32]^

## 3. Results

### 3.1. Causal effect of hypothyroidism on OSA (forward direction MR)

The IVW method showed that there was a causal effect of hypothyroidism on OSA (odds ratio, 1.870 [95% confidence interval, 1.055–3.315]; *P* = .032; Table 2). The other 4 supplementary MR methods, including the MR-Egger regression, weighted median, weighted mode, and simple mode, also showed consistent results.

**Table 2:** MR estimates of the relationship between hypothyroidism and OSA.

Exposure	Outcome	Method	nSNP	B	OR	95% CI	*P* value
Hypothyroidism	OSA	MR-Egger	120	0.558935997	1.74881077	(0.508168213–6.018359725)	.377191226
		Weighted median		0.70521405	2.024279936	(0.803023913–5.102848362)	.134924263
		Inverse variance weighted		0.626098934	1.870300165	(1.055146226–3.315201838)	.032048799
		Simple mode		0.422733464	1.526127474	(0.222100709–10.48652695)	.668050949
		Weighted mode		0.659911581	1.93462127	(0.694133269–5.39198973)	.209461066
OSA	Hypothyroidism	MR-Egger	5	−0.026501962	0.973846133	(0.943202063–1.005485811)	.202708252
		Weighted median		−0.001843172	0.998158525	(0.990995532–1.005373293)	.615943659
		Inverse variance weighted		0.000588085	1.000588258	(0.992934383–1.008301133)	.880679338
		Simple mode		−0.002836258	0.99716776	(0.986779579–1.007665302)	.62363927
		Weighted mode		−0.002231906	0.997770583	(0.989273062–1.006341095)	.635966667

CI = confidence interval, MR = Mendelian randomization, nSNP = number of single nucleotide polymorphism, OR = odds ratio, OSA = obstructive sleep apnea.

**Figure 3.**
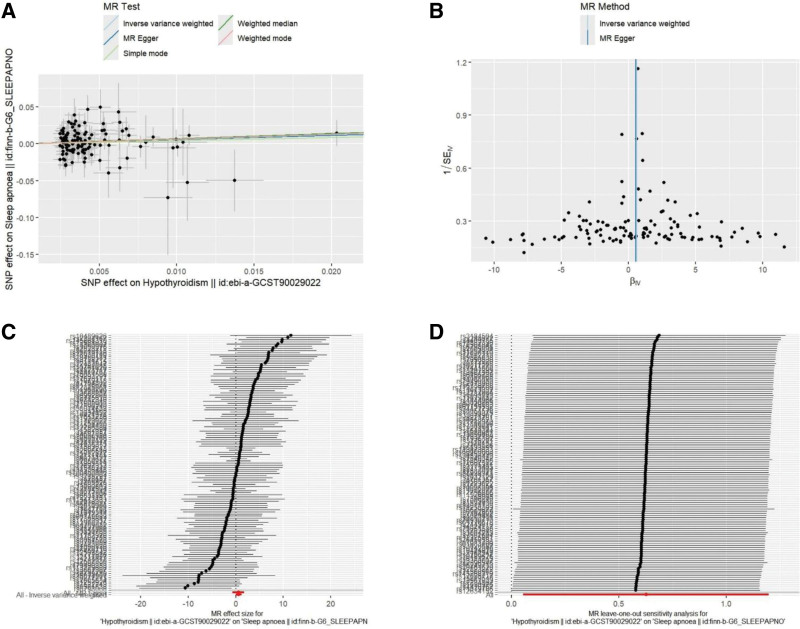
Visualization of Mendelian randomization (MR) estimates showing the causal effects of genetically predicted hypothyroidism on obstructive sleep apnea. (A) Scatter plot. (B) Funnel plot. (C) Forest plot. (D) Leave-one-out plot.

The results of the sensitivity analysis are listed in Table 3. No heterogeneity was observed among the selected IVs in the heterogeneity test. The MR-Egger regression and MR-PRESSO global tests showed no pleiotropy for the IVs. Furthermore, we found that no single SNP obviously drove or biased the overall effect in the leave-one-out sensitivity analysis. The leave-one-out plot, scatter plot, forest plot, and funnel plot are shown in Figure 3.

**Table 3:** Sensitivity analysis of the relationship between hypothyroidism and OSA.

Exposure	Outcome	Pleiotropy		Heterogeneity		Outlier examination by MR-PRESSO	
		Horizontal pleiotropy (Egger intercept)	Horizontal pleiotropy (*P* value)	Heterogeneity (Q)	Heterogeneity (*P* value)	Before correction (*P* value)	After correction (*P* value)
Hypothyroidism	OSA	0.000344707	.904542451	116.7815161	.540386744	.01750697	NA
OSA	Hypothyroidism	0.002531148	.1887803	7.322445	.1197984	.8879443	NA

MR = Mendelian randomization, OSA = obstructive sleep apnea.

### 3.2. Causal effect of OSA on hypothyroidism (reverse direction MR)

**Figure 4.**
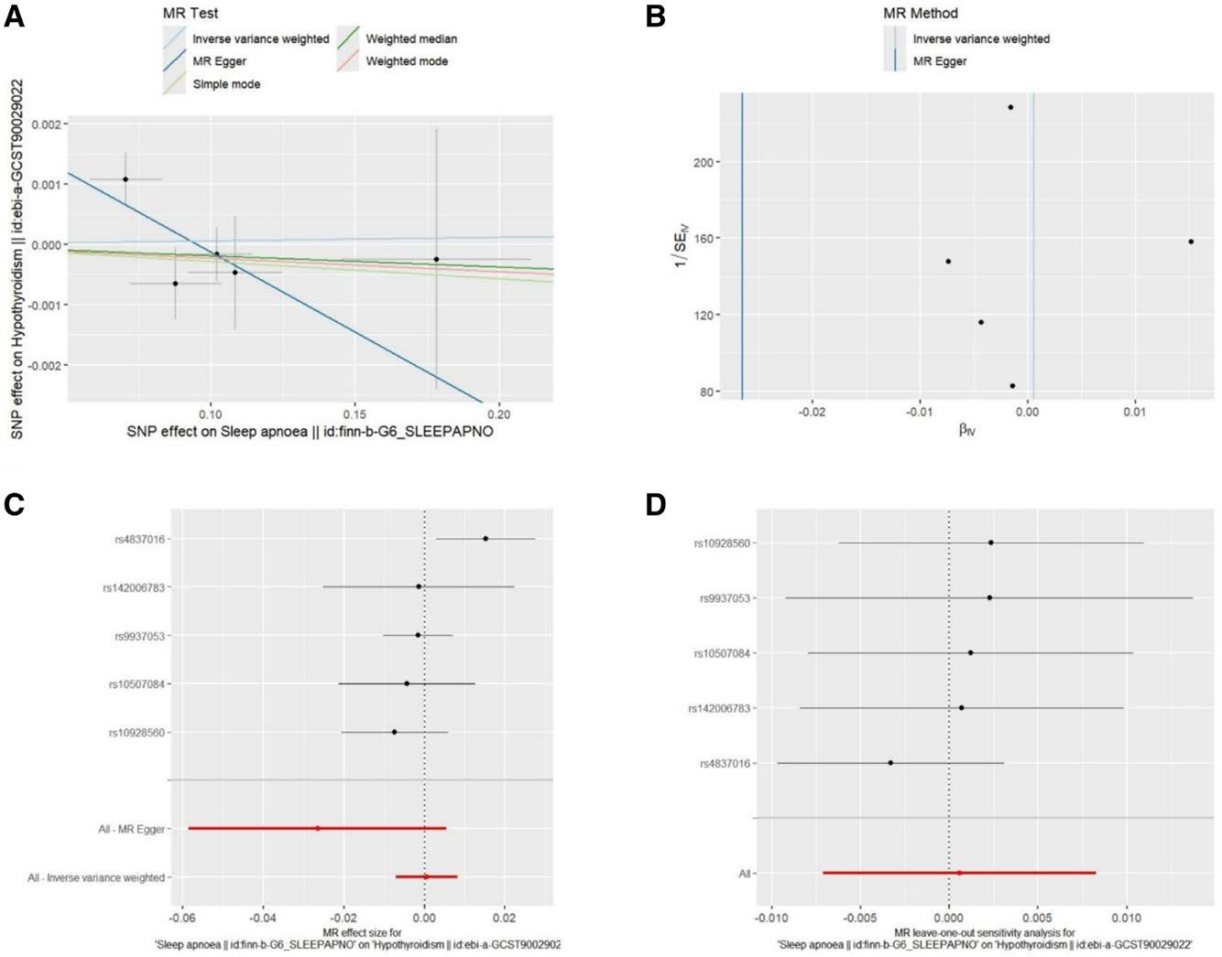
Visualization of Mendelian randomization (MR) estimates showing the causal effects of genetically predicted obstructive sleep apnea on hypothyroidism. (A) Scatter plot. (B) Funnel plot. (C) Forest plot. (D) Leave-one-out plot.

The primary analysis showed that there was no evidence to support a causal relationship of OSA on hypothyroidism (odds ratio, 1.001 [95% confidence interval, 0.993–1.008]; *P* = .881; Table 2). In the sensitivity analysis (Table 3), no heterogeneity or pleiotropy was found in the valid IVs. The leave-one-out plot, scatter plot, forest plot, and funnel plot are shown in Figure 4.

## 4. Discussion

To the best of our knowledge, this is the first MR study to explore causality between hypothyroidism and OSA. Our analysis revealed a significant positive causal effect of hypothyroidism on the risk of OSA.

At present, there is still inconsistency regarding the causal relationship between hypothyroidism and OSA. A cross-sectional study with a total of 5515 participants and a multivariate logistic regression analysis adjusting for confounding factors demonstrated a significant association between hypothyroidism and SA.^[33]^ Another cross-sectional study including 100 hypothyroidism patients done over a period of 1 year in a tertiary care hospital showed that the prevalence of OSA is quite high in hypothyroidism.^[34]^ A meta-analysis from 12 studies and 5 case reports showed that hypothyroidism is found to be associated with the severity of OSA.^[35]^ Contrary to these studies, a recent meta-analysis included 23 articles, which indicated a lower incidence of thyroid dysfunction in OSA-hypopnea syndrome individuals.^[36]^ A cross-sectional study including 813 patients found that the incidence of thyroid function disorders did not differ between patients with OSA and the general population.^[37]^

The observed causal effect may be explained by several physiological mechanisms, including constriction of the pharynx due to submucosal accumulation of mucopolysaccharides and protein, dysfunction of pharyngeal dilator muscles, and inhibition of the respiratory center.^[33,34]^ In addition, hypothyroidism is often associated with weight gain and obesity, which are significant risk factors for OSA.^[13,38]^

Our study had several obvious advantages, including the use of MR to infer causality and the large sample size provided by the publicly available GWAS datasets. Furthermore, we applied strict criteria to select SNPs and a series of sensitivity analyses to further test the robustness and reliability of our results.

Despite the strengths of this study, it has several limitations. First, the GWAS dataset obtained in our study was not recently published. In addition, our study focused on populations of European ancestry, and the generalizability of our findings to other ethnic groups requires further investigation. Therefore, further research is needed to validate our results.

## 5. Conclusion

Our study showed that hypothyroidism was causally associated with an increased risk of OSA, and no causality of genetically predicted OSA with the risk of hypothyroidism was observed.

## Acknowledgments

The authors thank all colleagues and staff for sharing genome-wide association study summary statistics.

## Author contributions

**Data curation:** Ning Lu, Zhaojun Lu.

**Formal analysis:** Ning Lu, Zhaojun Lu.

**Methodology:** Ning Lu.

**Project administration:** Ning Lu.

**Software:** Ning Lu, Cuocuo Wang.

**Writing – original draft:** Ning Lu.

**Conceptualization:** Bi Chen, Zhaojun Lu, Shengli Li.

**Funding acquisition:** Bi Chen.

**Resources:** Bi Chen.

**Investigation:** Pingli Liu.

**Validation:** Pingli Liu.

**Visualization:** Pingli Liu.

**Supervision:** Cuocuo Wang.

**Writing – review & editing:** Shengli Li.

## Supplementary Material


