# Supplementary material for: Causal association between hypothyroidism and obstructive sleep apnea: A bidirectional 2-sample Mendelian Randomization study
Source: Medicine (Baltimore). 2024 Oct 18;103(42):e40114. doi: 10.1097/MD.0000000000040114 (PMC11495703; doi:10.1097/MD.0000000000040114)
Supplement: Supplementary file 1 [file medi-103-e40114-s001.docx]

| Supplementary Table 1 SNPs used as valid instrumental variables for hypothyroidism on OSA. | | | | | | | | | | | | | | | | | | | | | | | | | | | | | | | | | | | | |
| --- | --- | --- | --- | --- | --- | --- | --- | --- | --- | --- | --- | --- | --- | --- | --- | --- | --- | --- | --- | --- | --- | --- | --- | --- | --- | --- | --- | --- | --- | --- | --- | --- | --- | --- | --- | --- |
| SNP | effect_allele.exposure | other_allele.exposure | effect_allele.outcome | other_allele.outcome | beta.exposure | beta.outcome | eaf.exposure | eaf.outcome | remove | palindromic | ambiguous | id.outcome | chr | pos | se.outcome | samplesize.outcome | pval.outcome | outcome | originalname.outcome | outcome.deprecated | mr_keep.outcome | data_source.outcome | chr.exposure | pos.exposure | se.exposure | pval.exposure | samplesize.exposure | id.exposure | exposure | mr_keep.exposure | pval_origin.exposure | data_source.exposure | action | SNP_index | mr_keep | F |
| rs10036386 | T | C | T | C | 0.0032806 | 0.0044 | 0.38175 | 0.4177 | FALSE | FALSE | FALSE | finn-b-G6_SLEEPAPNO | 5 | 76543603 | 0.0126 |  | 0.726599 | Sleep apnoea \|\| id:finn-b-G6_SLEEPAPNO | Sleep apnoea | Sleep apnoea \|\| \|\| | TRUE | igd | 5 | 76543603 | 0.00045434 | 1.8001E-13 | 473703 | ebi-a-GCST90029022 | Hypothyroidism \|\| id:ebi-a-GCST90029022 | TRUE | reported | igd | 2 | 1 | TRUE | 52.13751755 |
| rs1005048 | T | C | T | C | -0.0025135 | -0.0077 | 0.405976 | 0.3756 | FALSE | FALSE | FALSE | finn-b-G6_SLEEPAPNO | 12 | 68500239 | 0.0128 |  | 0.5465 | Sleep apnoea \|\| id:finn-b-G6_SLEEPAPNO | Sleep apnoea | Sleep apnoea \|\| \|\| | TRUE | igd | 12 | 68500239 | 0.00044895 | 1.8E-08 | 473703 | ebi-a-GCST90029022 | Hypothyroidism \|\| id:ebi-a-GCST90029022 | TRUE | reported | igd | 2 | 1 | TRUE | 31.34567199 |
| rs10277273 | G | T | G | T | -0.0026783 | 0.0208 | 0.570873 | 0.7038 | FALSE | FALSE | FALSE | finn-b-G6_SLEEPAPNO | 7 | 4785129 | 0.014 |  | 0.1377 | Sleep apnoea \|\| id:finn-b-G6_SLEEPAPNO | Sleep apnoea | Sleep apnoea \|\| \|\| | TRUE | igd | 7 | 4785129 | 0.00045098 | 5.6999E-10 | 473703 | ebi-a-GCST90029022 | Hypothyroidism \|\| id:ebi-a-GCST90029022 | TRUE | reported | igd | 2 | 1 | TRUE | 35.26966569 |
| rs1032129 | C | A | C | A | -0.0029182 | -0.0035 | 0.35562 | 0.4088 | FALSE | FALSE | FALSE | finn-b-G6_SLEEPAPNO | 8 | 119951900 | 0.0127 |  | 0.7842 | Sleep apnoea \|\| id:finn-b-G6_SLEEPAPNO | Sleep apnoea | Sleep apnoea \|\| \|\| | TRUE | igd | 8 | 119951900 | 0.00046223 | 1.9E-10 | 473703 | ebi-a-GCST90029022 | Hypothyroidism \|\| id:ebi-a-GCST90029022 | TRUE | reported | igd | 2 | 1 | TRUE | 39.85856695 |
| rs10424978 | A | C | A | C | -0.0045173 | -0.0117 | 0.600063 | 0.5992 | FALSE | FALSE | FALSE | finn-b-G6_SLEEPAPNO | 19 | 4837557 | 0.0127 |  | 0.3572 | Sleep apnoea \|\| id:finn-b-G6_SLEEPAPNO | Sleep apnoea | Sleep apnoea \|\| \|\| | TRUE | igd | 19 | 4837557 | 0.00045546 | 1.5E-23 | 473703 | ebi-a-GCST90029022 | Hypothyroidism \|\| id:ebi-a-GCST90029022 | TRUE | reported | igd | 2 | 1 | TRUE | 98.37010173 |
| rs10489626 | G | C | G | C | 0.00337558 | 0.0392 | 0.164184 | 0.08773 | FALSE | TRUE | FALSE | finn-b-G6_SLEEPAPNO | 1 | 67793171 | 0.022 |  | 0.0753894 | Sleep apnoea \|\| id:finn-b-G6_SLEEPAPNO | Sleep apnoea | Sleep apnoea \|\| \|\| | TRUE | igd | 1 | 67793171 | 0.00059576 | 4.7E-09 | 473703 | ebi-a-GCST90029022 | Hypothyroidism \|\| id:ebi-a-GCST90029022 | TRUE | reported | igd | 2 | 1 | TRUE | 32.10416792 |
| rs1050976 | T | C | T | C | 0.00320235 | 0.0272 | 0.528951 | 0.4729 | FALSE | FALSE | FALSE | finn-b-G6_SLEEPAPNO | 6 | 408079 | 0.0124 |  | 0.0285503 | Sleep apnoea \|\| id:finn-b-G6_SLEEPAPNO | Sleep apnoea | Sleep apnoea \|\| \|\| | TRUE | igd | 6 | 408079 | 0.00044375 | 3.8001E-13 | 473703 | ebi-a-GCST90029022 | Hypothyroidism \|\| id:ebi-a-GCST90029022 | TRUE | reported | igd | 2 | 1 | TRUE | 52.07925647 |
| rs10742340 | C | T | C | T | 0.00404692 | 0.0127 | 0.38757 | 0.3663 | FALSE | FALSE | FALSE | finn-b-G6_SLEEPAPNO | 11 | 35317712 | 0.0129 |  | 0.3248 | Sleep apnoea \|\| id:finn-b-G6_SLEEPAPNO | Sleep apnoea | Sleep apnoea \|\| \|\| | TRUE | igd | 11 | 35317712 | 0.00045489 | 1.1E-18 | 473703 | ebi-a-GCST90029022 | Hypothyroidism \|\| id:ebi-a-GCST90029022 | TRUE | reported | igd | 2 | 1 | TRUE | 79.14806069 |
| rs10761620 | G | A | G | A | -0.0034206 | -0.0181 | 0.549016 | 0.6261 | FALSE | FALSE | FALSE | finn-b-G6_SLEEPAPNO | 10 | 64057202 | 0.0129 |  | 0.1606 | Sleep apnoea \|\| id:finn-b-G6_SLEEPAPNO | Sleep apnoea | Sleep apnoea \|\| \|\| | TRUE | igd | 10 | 64057202 | 0.00044535 | 5.6002E-15 | 473703 | ebi-a-GCST90029022 | Hypothyroidism \|\| id:ebi-a-GCST90029022 | TRUE | reported | igd | 2 | 1 | TRUE | 58.99414102 |
| rs1079418 | G | A | G | A | -0.0029149 | -0.0122 | 0.306362 | 0.2626 | FALSE | FALSE | FALSE | finn-b-G6_SLEEPAPNO | 6 | 166047034 | 0.0141 |  | 0.3865 | Sleep apnoea \|\| id:finn-b-G6_SLEEPAPNO | Sleep apnoea | Sleep apnoea \|\| \|\| | TRUE | igd | 6 | 166047034 | 0.00047833 | 5.1E-10 | 473703 | ebi-a-GCST90029022 | Hypothyroidism \|\| id:ebi-a-GCST90029022 | TRUE | reported | igd | 2 | 1 | TRUE | 37.13600661 |
| rs1088898 | T | G | T | G | 0.00287708 | -0.0138 | 0.769 | 0.6971 | FALSE | FALSE | FALSE | finn-b-G6_SLEEPAPNO | 17 | 8876505 | 0.0136 |  | 0.3073 | Sleep apnoea \|\| id:finn-b-G6_SLEEPAPNO | Sleep apnoea | Sleep apnoea \|\| \|\| | TRUE | igd | 17 | 8876505 | 0.00052677 | 4.4E-08 | 473703 | ebi-a-GCST90029022 | Hypothyroidism \|\| id:ebi-a-GCST90029022 | TRUE | reported | igd | 2 | 1 | TRUE | 29.8304744 |
| rs11052877 | G | A | G | A | -0.005342 | -0.0048 | 0.371549 | 0.4098 | FALSE | FALSE | FALSE | finn-b-G6_SLEEPAPNO | 12 | 9905690 | 0.0127 |  | 0.7021 | Sleep apnoea \|\| id:finn-b-G6_SLEEPAPNO | Sleep apnoea | Sleep apnoea \|\| \|\| | TRUE | igd | 12 | 9905690 | 0.00045769 | 4.1995E-32 | 473703 | ebi-a-GCST90029022 | Hypothyroidism \|\| id:ebi-a-GCST90029022 | TRUE | reported | igd | 2 | 1 | TRUE | 136.2285619 |
| rs11073337 | C | A | C | A | 0.00413065 | -0.0078 | 0.251826 | 0.2623 | FALSE | FALSE | FALSE | finn-b-G6_SLEEPAPNO | 15 | 38847763 | 0.0141 |  | 0.5814 | Sleep apnoea \|\| id:finn-b-G6_SLEEPAPNO | Sleep apnoea | Sleep apnoea \|\| \|\| | TRUE | igd | 15 | 38847763 | 0.00050987 | 6.0007E-17 | 473703 | ebi-a-GCST90029022 | Hypothyroidism \|\| id:ebi-a-GCST90029022 | TRUE | reported | igd | 2 | 1 | TRUE | 65.63233818 |
| rs11079786 | A | G | A | G | 0.00405211 | 0.0281 | 0.257926 | 0.2926 | FALSE | FALSE | FALSE | finn-b-G6_SLEEPAPNO | 17 | 45805916 | 0.0137 |  | 0.0399301 | Sleep apnoea \|\| id:finn-b-G6_SLEEPAPNO | Sleep apnoea | Sleep apnoea \|\| \|\| | TRUE | igd | 17 | 45805916 | 0.00050412 | 7.3995E-16 | 473703 | ebi-a-GCST90029022 | Hypothyroidism \|\| id:ebi-a-GCST90029022 | TRUE | reported | igd | 2 | 1 | TRUE | 64.61051645 |
| rs11258303 | A | C | A | C | 0.00365252 | 0.0072 | 0.746348 | 0.6542 | FALSE | FALSE | FALSE | finn-b-G6_SLEEPAPNO | 10 | 6405534 | 0.0131 |  | 0.585 | Sleep apnoea \|\| id:finn-b-G6_SLEEPAPNO | Sleep apnoea | Sleep apnoea \|\| \|\| | TRUE | igd | 10 | 6405534 | 0.00050869 | 2.0999E-12 | 473703 | ebi-a-GCST90029022 | Hypothyroidism \|\| id:ebi-a-GCST90029022 | TRUE | reported | igd | 2 | 1 | TRUE | 51.55534322 |
| rs113229608 | A | C | A | C | 0.00631459 | -0.0326 | 0.063917 | 0.03793 | FALSE | FALSE | FALSE | finn-b-G6_SLEEPAPNO | 3 | 121743159 | 0.0324 |  | 0.3149 | Sleep apnoea \|\| id:finn-b-G6_SLEEPAPNO | Sleep apnoea | Sleep apnoea \|\| \|\| | TRUE | igd | 3 | 121743159 | 0.00090606 | 2.9E-12 | 473703 | ebi-a-GCST90029022 | Hypothyroidism \|\| id:ebi-a-GCST90029022 | TRUE | reported | igd | 2 | 1 | TRUE | 48.57082055 |
| rs113473633 | G | A | G | A | -0.0097711 | 0.0057 | 0.025981 | 0.0138 | FALSE | FALSE | FALSE | finn-b-G6_SLEEPAPNO | 4 | 103449131 | 0.0544 |  | 0.916 | Sleep apnoea \|\| id:finn-b-G6_SLEEPAPNO | Sleep apnoea | Sleep apnoea \|\| \|\| | TRUE | igd | 4 | 103449131 | 0.00144267 | 2.2999E-11 | 473703 | ebi-a-GCST90029022 | Hypothyroidism \|\| id:ebi-a-GCST90029022 | TRUE | reported | igd | 2 | 1 | TRUE | 45.87227166 |
| rs11675342 | T | C | T | C | 0.00505693 | -0.0024 | 0.423445 | 0.442 | FALSE | FALSE | FALSE | finn-b-G6_SLEEPAPNO | 2 | 1407628 | 0.0126 |  | 0.8488 | Sleep apnoea \|\| id:finn-b-G6_SLEEPAPNO | Sleep apnoea | Sleep apnoea \|\| \|\| | TRUE | igd | 2 | 1407628 | 0.00044658 | 8.6996E-30 | 473703 | ebi-a-GCST90029022 | Hypothyroidism \|\| id:ebi-a-GCST90029022 | TRUE | reported | igd | 2 | 1 | TRUE | 128.226353 |
| rs11782370 | T | C | T | C | -0.002939 | 0.0084 | 0.239468 | 0.1594 | FALSE | FALSE | FALSE | finn-b-G6_SLEEPAPNO | 8 | 23370018 | 0.0171 |  | 0.6234 | Sleep apnoea \|\| id:finn-b-G6_SLEEPAPNO | Sleep apnoea | Sleep apnoea \|\| \|\| | TRUE | igd | 8 | 23370018 | 0.00051787 | 2.4E-08 | 473703 | ebi-a-GCST90029022 | Hypothyroidism \|\| id:ebi-a-GCST90029022 | TRUE | reported | igd | 2 | 1 | TRUE | 32.20826494 |
| rs11926659 | G | A | G | A | -0.0036194 | -0.0083 | 0.136373 | 0.1805 | FALSE | FALSE | FALSE | finn-b-G6_SLEEPAPNO | 3 | 105499839 | 0.0162 |  | 0.608199 | Sleep apnoea \|\| id:finn-b-G6_SLEEPAPNO | Sleep apnoea | Sleep apnoea \|\| \|\| | TRUE | igd | 3 | 105499839 | 0.00064454 | 0.00000001 | 473703 | ebi-a-GCST90029022 | Hypothyroidism \|\| id:ebi-a-GCST90029022 | TRUE | reported | igd | 2 | 1 | TRUE | 31.53291432 |
| rs12117927 | A | C | A | C | 0.0026691 | -0.0104 | 0.488931 | 0.4022 | FALSE | FALSE | FALSE | finn-b-G6_SLEEPAPNO | 1 | 236629134 | 0.0129 |  | 0.4227 | Sleep apnoea \|\| id:finn-b-G6_SLEEPAPNO | Sleep apnoea | Sleep apnoea \|\| \|\| | TRUE | igd | 1 | 236629134 | 0.00045206 | 1.1E-09 | 473703 | ebi-a-GCST90029022 | Hypothyroidism \|\| id:ebi-a-GCST90029022 | TRUE | reported | igd | 2 | 1 | TRUE | 34.86050598 |
| rs12271161 | A | G | A | G | -0.0036517 | 0.0021 | 0.193356 | 0.2819 | FALSE | FALSE | FALSE | finn-b-G6_SLEEPAPNO | 11 | 116979911 | 0.0139 |  | 0.877 | Sleep apnoea \|\| id:finn-b-G6_SLEEPAPNO | Sleep apnoea | Sleep apnoea \|\| \|\| | TRUE | igd | 11 | 116979911 | 0.00055896 | 3.1003E-11 | 473703 | ebi-a-GCST90029022 | Hypothyroidism \|\| id:ebi-a-GCST90029022 | TRUE | reported | igd | 2 | 1 | TRUE | 42.67956038 |
| rs12325861 | C | T | C | T | 0.00415495 | 0.0153 | 0.165637 | 0.2028 | FALSE | FALSE | FALSE | finn-b-G6_SLEEPAPNO | 17 | 40289412 | 0.0155 |  | 0.3224 | Sleep apnoea \|\| id:finn-b-G6_SLEEPAPNO | Sleep apnoea | Sleep apnoea \|\| \|\| | TRUE | igd | 17 | 40289412 | 0.00059302 | 1.9002E-12 | 473703 | ebi-a-GCST90029022 | Hypothyroidism \|\| id:ebi-a-GCST90029022 | TRUE | reported | igd | 2 | 1 | TRUE | 49.08982209 |
| rs12482947 | C | T | C | T | 0.00267033 | 0.0166 | 0.600482 | 0.5782 | FALSE | FALSE | FALSE | finn-b-G6_SLEEPAPNO | 21 | 43852037 | 0.0126 |  | 0.1877 | Sleep apnoea \|\| id:finn-b-G6_SLEEPAPNO | Sleep apnoea | Sleep apnoea \|\| \|\| | TRUE | igd | 21 | 43852037 | 0.00045376 | 1.8E-09 | 473703 | ebi-a-GCST90029022 | Hypothyroidism \|\| id:ebi-a-GCST90029022 | TRUE | reported | igd | 2 | 1 | TRUE | 34.63199032 |
| rs1257926 | A | G | A | G | 0.00277858 | -0.0219 | 0.47514 | 0.5559 | FALSE | FALSE | FALSE | finn-b-G6_SLEEPAPNO | 14 | 98692996 | 0.0125 |  | 0.0801807 | Sleep apnoea \|\| id:finn-b-G6_SLEEPAPNO | Sleep apnoea | Sleep apnoea \|\| \|\| | TRUE | igd | 14 | 98692996 | 0.00044275 | 1.1E-10 | 473703 | ebi-a-GCST90029022 | Hypothyroidism \|\| id:ebi-a-GCST90029022 | TRUE | reported | igd | 2 | 1 | TRUE | 39.38409078 |
| rs12582330 | T | G | T | G | -0.0040851 | 0.0195 | 0.728077 | 0.6738 | FALSE | FALSE | FALSE | finn-b-G6_SLEEPAPNO | 12 | 103892941 | 0.0134 |  | 0.1445 | Sleep apnoea \|\| id:finn-b-G6_SLEEPAPNO | Sleep apnoea | Sleep apnoea \|\| \|\| | TRUE | igd | 12 | 103892941 | 0.00049711 | 1.6998E-16 | 473703 | ebi-a-GCST90029022 | Hypothyroidism \|\| id:ebi-a-GCST90029022 | TRUE | reported | igd | 2 | 1 | TRUE | 67.52984512 |
| rs12634152 | T | C | T | C | -0.0064934 | -0.0173 | 0.547336 | 0.4555 | FALSE | FALSE | FALSE | finn-b-G6_SLEEPAPNO | 3 | 188121019 | 0.0125 |  | 0.1662 | Sleep apnoea \|\| id:finn-b-G6_SLEEPAPNO | Sleep apnoea | Sleep apnoea \|\| \|\| | TRUE | igd | 3 | 188121019 | 0.0004439 | 1.5999E-49 | 473703 | ebi-a-GCST90029022 | Hypothyroidism \|\| id:ebi-a-GCST90029022 | TRUE | reported | igd | 2 | 1 | TRUE | 213.9774726 |
| rs12697352 | A | G | A | G | -0.0026674 | -0.0014 | 0.340258 | 0.395 | FALSE | FALSE | FALSE | finn-b-G6_SLEEPAPNO | 5 | 35837234 | 0.0128 |  | 0.9128 | Sleep apnoea \|\| id:finn-b-G6_SLEEPAPNO | Sleep apnoea | Sleep apnoea \|\| \|\| | TRUE | igd | 5 | 35837234 | 0.00046622 | 6.6999E-09 | 473703 | ebi-a-GCST90029022 | Hypothyroidism \|\| id:ebi-a-GCST90029022 | TRUE | reported | igd | 2 | 1 | TRUE | 32.7346362 |
| rs12756886 | C | T | C | T | 0.00436854 | 0.0071 | 0.120141 | 0.1308 | FALSE | FALSE | FALSE | finn-b-G6_SLEEPAPNO | 1 | 200840467 | 0.0184 |  | 0.7005 | Sleep apnoea \|\| id:finn-b-G6_SLEEPAPNO | Sleep apnoea | Sleep apnoea \|\| \|\| | TRUE | igd | 1 | 200840467 | 0.00067955 | 9.3994E-11 | 473703 | ebi-a-GCST90029022 | Hypothyroidism \|\| id:ebi-a-GCST90029022 | TRUE | reported | igd | 2 | 1 | TRUE | 41.32624681 |
| rs12981033 | G | A | G | A | -0.0030302 | -0.0293 | 0.392988 | 0.3572 | FALSE | FALSE | FALSE | finn-b-G6_SLEEPAPNO | 19 | 50197406 | 0.013 |  | 0.0242003 | Sleep apnoea \|\| id:finn-b-G6_SLEEPAPNO | Sleep apnoea | Sleep apnoea \|\| \|\| | TRUE | igd | 19 | 50197406 | 0.00045235 | 7E-12 | 473703 | ebi-a-GCST90029022 | Hypothyroidism \|\| id:ebi-a-GCST90029022 | TRUE | reported | igd | 2 | 1 | TRUE | 44.87544128 |
| rs13090803 | T | G | T | G | 0.00469389 | -0.0017 | 0.212253 | 0.1386 | FALSE | FALSE | FALSE | finn-b-G6_SLEEPAPNO | 3 | 105934953 | 0.018 |  | 0.9257 | Sleep apnoea \|\| id:finn-b-G6_SLEEPAPNO | Sleep apnoea | Sleep apnoea \|\| \|\| | TRUE | igd | 3 | 105934953 | 0.00054339 | 1.8001E-18 | 473703 | ebi-a-GCST90029022 | Hypothyroidism \|\| id:ebi-a-GCST90029022 | TRUE | reported | igd | 2 | 1 | TRUE | 74.6167172 |
| rs13297295 | C | T | C | T | -0.0040226 | 0.0251 | 0.11951 | 0.1049 | FALSE | FALSE | FALSE | finn-b-G6_SLEEPAPNO | 9 | 131659724 | 0.0203 |  | 0.2162 | Sleep apnoea \|\| id:finn-b-G6_SLEEPAPNO | Sleep apnoea | Sleep apnoea \|\| \|\| | TRUE | igd | 9 | 131659724 | 0.00068523 | 3.1E-09 | 473703 | ebi-a-GCST90029022 | Hypothyroidism \|\| id:ebi-a-GCST90029022 | TRUE | reported | igd | 2 | 1 | TRUE | 34.46128224 |
| rs13360007 | G | A | G | A | 0.00348628 | 0.0309 | 0.135608 | 0.1211 | FALSE | FALSE | FALSE | finn-b-G6_SLEEPAPNO | 5 | 156577720 | 0.0191 |  | 0.1051 | Sleep apnoea \|\| id:finn-b-G6_SLEEPAPNO | Sleep apnoea | Sleep apnoea \|\| \|\| | TRUE | igd | 5 | 156577720 | 0.00064449 | 1.8E-08 | 473703 | ebi-a-GCST90029022 | Hypothyroidism \|\| id:ebi-a-GCST90029022 | TRUE | reported | igd | 2 | 1 | TRUE | 29.26102042 |
| rs13398375 | C | T | C | T | -0.0028879 | 0.0059 | 0.292043 | 0.3482 | FALSE | FALSE | FALSE | finn-b-G6_SLEEPAPNO | 2 | 8451701 | 0.0131 |  | 0.6498 | Sleep apnoea \|\| id:finn-b-G6_SLEEPAPNO | Sleep apnoea | Sleep apnoea \|\| \|\| | TRUE | igd | 2 | 8451701 | 0.00048876 | 2.8E-09 | 473703 | ebi-a-GCST90029022 | Hypothyroidism \|\| id:ebi-a-GCST90029022 | TRUE | reported | igd | 2 | 1 | TRUE | 34.91220085 |
| rs138453996 | A | G | A | G | 0.0104088 | 0.0018 | 0.02009 | 0.01949 | FALSE | FALSE | FALSE | finn-b-G6_SLEEPAPNO | 16 | 67349478 | 0.0452 |  | 0.9688 | Sleep apnoea \|\| id:finn-b-G6_SLEEPAPNO | Sleep apnoea | Sleep apnoea \|\| \|\| | TRUE | igd | 16 | 67349478 | 0.00157772 | 3.1003E-11 | 473703 | ebi-a-GCST90029022 | Hypothyroidism \|\| id:ebi-a-GCST90029022 | TRUE | reported | igd | 2 | 1 | TRUE | 43.5252692 |
| rs145268310 | C | G | C | G | 0.00505679 | 0.0495 | 0.120794 | 0.0735 | FALSE | TRUE | FALSE | finn-b-G6_SLEEPAPNO | 3 | 12310773 | 0.0238 |  | 0.0377303 | Sleep apnoea \|\| id:finn-b-G6_SLEEPAPNO | Sleep apnoea | Sleep apnoea \|\| \|\| | TRUE | igd | 3 | 12310773 | 0.00067779 | 4.4999E-14 | 473703 | ebi-a-GCST90029022 | Hypothyroidism \|\| id:ebi-a-GCST90029022 | TRUE | reported | igd | 2 | 1 | TRUE | 55.66209176 |
| rs1534430 | T | C | T | C | -0.0037771 | -0.0064 | 0.390309 | 0.4174 | FALSE | FALSE | FALSE | finn-b-G6_SLEEPAPNO | 2 | 12644736 | 0.0126 |  | 0.612999 | Sleep apnoea \|\| id:finn-b-G6_SLEEPAPNO | Sleep apnoea | Sleep apnoea \|\| \|\| | TRUE | igd | 2 | 12644736 | 0.00045256 | 4.4005E-17 | 473703 | ebi-a-GCST90029022 | Hypothyroidism \|\| id:ebi-a-GCST90029022 | TRUE | reported | igd | 2 | 1 | TRUE | 69.65648805 |
| rs1611236 | A | G | A | G | -0.0054237 | -0.0133 | 0.323762 | 0.2233 | FALSE | FALSE | FALSE | finn-b-G6_SLEEPAPNO | 6 | 29748690 | 0.0154 |  | 0.3899 | Sleep apnoea \|\| id:finn-b-G6_SLEEPAPNO | Sleep apnoea | Sleep apnoea \|\| \|\| | TRUE | igd | 6 | 29748690 | 0.00086927 | 2.1E-10 | 473703 | ebi-a-GCST90029022 | Hypothyroidism \|\| id:ebi-a-GCST90029022 | TRUE | reported | igd | 2 | 1 | TRUE | 38.92949166 |
| rs17020110 | C | T | C | T | 0.00449604 | -0.0151 | 0.267479 | 0.2266 | FALSE | FALSE | FALSE | finn-b-G6_SLEEPAPNO | 1 | 108354156 | 0.0148 |  | 0.309 | Sleep apnoea \|\| id:finn-b-G6_SLEEPAPNO | Sleep apnoea | Sleep apnoea \|\| \|\| | TRUE | igd | 1 | 108354156 | 0.00049956 | 3.1003E-19 | 473703 | ebi-a-GCST90029022 | Hypothyroidism \|\| id:ebi-a-GCST90029022 | TRUE | reported | igd | 2 | 1 | TRUE | 81.00032429 |
| rs17025117 | C | A | C | A | -0.006326 | -0.0291 | 0.212432 | 0.1247 | FALSE | FALSE | FALSE | finn-b-G6_SLEEPAPNO | 4 | 149665827 | 0.019 |  | 0.1267 | Sleep apnoea \|\| id:finn-b-G6_SLEEPAPNO | Sleep apnoea | Sleep apnoea \|\| \|\| | TRUE | igd | 4 | 149665827 | 0.00053949 | 2.0999E-32 | 473703 | ebi-a-GCST90029022 | Hypothyroidism \|\| id:ebi-a-GCST90029022 | TRUE | reported | igd | 2 | 1 | TRUE | 137.4937208 |
| rs1723022 | T | G | T | G | 0.00263973 | 0.0055 | 0.372773 | 0.2202 | FALSE | FALSE | FALSE | finn-b-G6_SLEEPAPNO | 1 | 167405418 | 0.015 |  | 0.716101 | Sleep apnoea \|\| id:finn-b-G6_SLEEPAPNO | Sleep apnoea | Sleep apnoea \|\| \|\| | TRUE | igd | 1 | 167405418 | 0.00045995 | 5.3001E-09 | 473703 | ebi-a-GCST90029022 | Hypothyroidism \|\| id:ebi-a-GCST90029022 | TRUE | reported | igd | 2 | 1 | TRUE | 32.93789808 |
| rs17385641 | C | G | C | G | 0.00264507 | -0.0049 | 0.286581 | 0.2594 | FALSE | TRUE | FALSE | finn-b-G6_SLEEPAPNO | 7 | 77362489 | 0.0142 |  | 0.7289 | Sleep apnoea \|\| id:finn-b-G6_SLEEPAPNO | Sleep apnoea | Sleep apnoea \|\| \|\| | TRUE | igd | 7 | 77362489 | 0.00048803 | 0.00000002 | 473703 | ebi-a-GCST90029022 | Hypothyroidism \|\| id:ebi-a-GCST90029022 | TRUE | reported | igd | 2 | 1 | TRUE | 29.37559283 |
| rs174599 | C | G | C | G | -0.003019 | 0.0109 | 0.37583 | 0.4264 | FALSE | TRUE | TRUE | finn-b-G6_SLEEPAPNO | 11 | 61621556 | 0.0126 |  | 0.3868 | Sleep apnoea \|\| id:finn-b-G6_SLEEPAPNO | Sleep apnoea | Sleep apnoea \|\| \|\| | TRUE | igd | 11 | 61621556 | 0.00045623 | 1.1E-11 | 473703 | ebi-a-GCST90029022 | Hypothyroidism \|\| id:ebi-a-GCST90029022 | TRUE | reported | igd | 2 | 1 | FALSE | 43.78815895 |
| rs1782648 | A | G | A | G | 0.00247594 | 0.0135 | 0.368189 | 0.4254 | FALSE | FALSE | FALSE | finn-b-G6_SLEEPAPNO | 10 | 81060829 | 0.0126 |  | 0.2838 | Sleep apnoea \|\| id:finn-b-G6_SLEEPAPNO | Sleep apnoea | Sleep apnoea \|\| \|\| | TRUE | igd | 10 | 81060829 | 0.00045978 | 3.8E-08 | 473703 | ebi-a-GCST90029022 | Hypothyroidism \|\| id:ebi-a-GCST90029022 | TRUE | reported | igd | 2 | 1 | TRUE | 28.99905587 |
| rs1810396 | G | A | G | A | -0.0038772 | 0.0037 | 0.689221 | 0.7889 | FALSE | FALSE | FALSE | finn-b-G6_SLEEPAPNO | 8 | 133918769 | 0.0153 |  | 0.8082 | Sleep apnoea \|\| id:finn-b-G6_SLEEPAPNO | Sleep apnoea | Sleep apnoea \|\| \|\| | TRUE | igd | 8 | 133918769 | 0.00047654 | 3.1996E-16 | 473703 | ebi-a-GCST90029022 | Hypothyroidism \|\| id:ebi-a-GCST90029022 | TRUE | reported | igd | 2 | 1 | TRUE | 66.19746102 |
| rs1921309 | A | T | A | T | 0.00260473 | 0.0034 | 0.49609 | 0.4344 | FALSE | TRUE | TRUE | finn-b-G6_SLEEPAPNO | 2 | 162048975 | 0.0125 |  | 0.7888 | Sleep apnoea \|\| id:finn-b-G6_SLEEPAPNO | Sleep apnoea | Sleep apnoea \|\| \|\| | TRUE | igd | 2 | 162048975 | 0.00044158 | 3.7E-09 | 473703 | ebi-a-GCST90029022 | Hypothyroidism \|\| id:ebi-a-GCST90029022 | TRUE | reported | igd | 2 | 1 | FALSE | 34.79481261 |
| rs1995311 | G | T | G | T | -0.0026765 | -0.0016 | 0.510924 | 0.4795 | FALSE | FALSE | FALSE | finn-b-G6_SLEEPAPNO | 1 | 19820119 | 0.0125 |  | 0.8999 | Sleep apnoea \|\| id:finn-b-G6_SLEEPAPNO | Sleep apnoea | Sleep apnoea \|\| \|\| | TRUE | igd | 1 | 19820119 | 0.00044149 | 4.2E-10 | 473703 | ebi-a-GCST90029022 | Hypothyroidism \|\| id:ebi-a-GCST90029022 | TRUE | reported | igd | 2 | 1 | TRUE | 36.75387879 |
| rs2111485 | G | A | G | A | 0.00382984 | 0.003 | 0.607345 | 0.5824 | FALSE | FALSE | FALSE | finn-b-G6_SLEEPAPNO | 2 | 163110536 | 0.0126 |  | 0.8117 | Sleep apnoea \|\| id:finn-b-G6_SLEEPAPNO | Sleep apnoea | Sleep apnoea \|\| \|\| | TRUE | igd | 2 | 163110536 | 0.00045114 | 1.8001E-17 | 473703 | ebi-a-GCST90029022 | Hypothyroidism \|\| id:ebi-a-GCST90029022 | TRUE | reported | igd | 2 | 1 | TRUE | 72.0660784 |
| rs221781 | G | A | G | A | 0.00437904 | -0.0045 | 0.88677 | 0.8475 | FALSE | FALSE | FALSE | finn-b-G6_SLEEPAPNO | 7 | 100295908 | 0.0173 |  | 0.794301 | Sleep apnoea \|\| id:finn-b-G6_SLEEPAPNO | Sleep apnoea | Sleep apnoea \|\| \|\| | TRUE | igd | 7 | 100295908 | 0.00069586 | 7.1007E-11 | 473703 | ebi-a-GCST90029022 | Hypothyroidism \|\| id:ebi-a-GCST90029022 | TRUE | reported | igd | 2 | 1 | TRUE | 39.6019504 |
| rs2234167 | A | G | A | G | 0.00426908 | 0.0468 | 0.133428 | 0.1214 | FALSE | FALSE | FALSE | finn-b-G6_SLEEPAPNO | 1 | 2494330 | 0.0191 |  | 0.0140602 | Sleep apnoea \|\| id:finn-b-G6_SLEEPAPNO | Sleep apnoea | Sleep apnoea \|\| \|\| | TRUE | igd | 1 | 2494330 | 0.00064736 | 2.2999E-11 | 473703 | ebi-a-GCST90029022 | Hypothyroidism \|\| id:ebi-a-GCST90029022 | TRUE | reported | igd | 2 | 1 | TRUE | 43.48820652 |
| rs229540 | G | T | G | T | 0.00518151 | 0.018 | 0.425127 | 0.3988 | FALSE | FALSE | FALSE | finn-b-G6_SLEEPAPNO | 22 | 37591290 | 0.0127 |  | 0.156 | Sleep apnoea \|\| id:finn-b-G6_SLEEPAPNO | Sleep apnoea | Sleep apnoea \|\| \|\| | TRUE | igd | 22 | 37591290 | 0.00044676 | 6.5993E-31 | 473703 | ebi-a-GCST90029022 | Hypothyroidism \|\| id:ebi-a-GCST90029022 | TRUE | reported | igd | 2 | 1 | TRUE | 134.512956 |
| rs2412974 | T | C | T | C | -0.0026243 | -0.003 | 0.360082 | 0.4032 | FALSE | FALSE | FALSE | finn-b-G6_SLEEPAPNO | 22 | 30539821 | 0.0127 |  | 0.8144 | Sleep apnoea \|\| id:finn-b-G6_SLEEPAPNO | Sleep apnoea | Sleep apnoea \|\| \|\| | TRUE | igd | 22 | 30539821 | 0.00045972 | 9.6E-09 | 473703 | ebi-a-GCST90029022 | Hypothyroidism \|\| id:ebi-a-GCST90029022 | TRUE | reported | igd | 2 | 1 | TRUE | 32.5856903 |
| rs2445610 | G | A | G | A | -0.003422 | -0.0109 | 0.368287 | 0.3397 | FALSE | FALSE | FALSE | finn-b-G6_SLEEPAPNO | 8 | 128197088 | 0.0131 |  | 0.4072 | Sleep apnoea \|\| id:finn-b-G6_SLEEPAPNO | Sleep apnoea | Sleep apnoea \|\| \|\| | TRUE | igd | 8 | 128197088 | 0.0004571 | 9.0991E-14 | 473703 | ebi-a-GCST90029022 | Hypothyroidism \|\| id:ebi-a-GCST90029022 | TRUE | reported | igd | 2 | 1 | TRUE | 56.0446815 |
| rs244672 | T | C | T | C | -0.0050429 | 0.0224 | 0.87565 | 0.7586 | FALSE | FALSE | FALSE | finn-b-G6_SLEEPAPNO | 5 | 133419283 | 0.0145 |  | 0.1238 | Sleep apnoea \|\| id:finn-b-G6_SLEEPAPNO | Sleep apnoea | Sleep apnoea \|\| \|\| | TRUE | igd | 5 | 133419283 | 0.00066873 | 7.1007E-14 | 473703 | ebi-a-GCST90029022 | Hypothyroidism \|\| id:ebi-a-GCST90029022 | TRUE | reported | igd | 2 | 1 | TRUE | 56.86723236 |
| rs2473808 | C | T | C | T | -0.0031858 | 0.0098 | 0.663277 | 0.6207 | FALSE | FALSE | FALSE | finn-b-G6_SLEEPAPNO | 1 | 19638883 | 0.0129 |  | 0.4462 | Sleep apnoea \|\| id:finn-b-G6_SLEEPAPNO | Sleep apnoea | Sleep apnoea \|\| \|\| | TRUE | igd | 1 | 19638883 | 0.00046761 | 9.0991E-12 | 473703 | ebi-a-GCST90029022 | Hypothyroidism \|\| id:ebi-a-GCST90029022 | TRUE | reported | igd | 2 | 1 | TRUE | 46.41601132 |
| rs2596546 | G | A | G | A | 0.0108008 | 0.0113 | 0.327209 | 0.3492 | FALSE | FALSE | FALSE | finn-b-G6_SLEEPAPNO | 6 | 31329386 | 0.0136 |  | 0.4058 | Sleep apnoea \|\| id:finn-b-G6_SLEEPAPNO | Sleep apnoea | Sleep apnoea \|\| \|\| | TRUE | igd | 6 | 31329386 | 0.00067283 | 1.6998E-47 | 473703 | ebi-a-GCST90029022 | Hypothyroidism \|\| id:ebi-a-GCST90029022 | TRUE | reported | igd | 2 | 1 | TRUE | 257.694427 |
| rs28157 | T | G | T | G | -0.0032879 | 0.0006 | 0.315388 | 0.3147 | FALSE | FALSE | FALSE | finn-b-G6_SLEEPAPNO | 5 | 102595837 | 0.0134 |  | 0.9672 | Sleep apnoea \|\| id:finn-b-G6_SLEEPAPNO | Sleep apnoea | Sleep apnoea \|\| \|\| | TRUE | igd | 5 | 102595837 | 0.00047578 | 5.3003E-12 | 473703 | ebi-a-GCST90029022 | Hypothyroidism \|\| id:ebi-a-GCST90029022 | TRUE | reported | igd | 2 | 1 | TRUE | 47.75614958 |
| rs2823272 | A | T | A | T | -0.003384 | -0.0267 | 0.315442 | 0.2566 | FALSE | TRUE | FALSE | finn-b-G6_SLEEPAPNO | 21 | 16798586 | 0.0143 |  | 0.0624799 | Sleep apnoea \|\| id:finn-b-G6_SLEEPAPNO | Sleep apnoea | Sleep apnoea \|\| \|\| | TRUE | igd | 21 | 16798586 | 0.00047592 | 7.1007E-13 | 473703 | ebi-a-GCST90029022 | Hypothyroidism \|\| id:ebi-a-GCST90029022 | TRUE | reported | igd | 2 | 1 | TRUE | 50.55774131 |
| rs28450181 | G | A | G | A | 0.00328649 | 0.0225 | 0.204036 | 0.1532 | FALSE | FALSE | FALSE | finn-b-G6_SLEEPAPNO | 4 | 87819369 | 0.0173 |  | 0.1933 | Sleep apnoea \|\| id:finn-b-G6_SLEEPAPNO | Sleep apnoea | Sleep apnoea \|\| \|\| | TRUE | igd | 4 | 87819369 | 0.00054894 | 1.7E-09 | 473703 | ebi-a-GCST90029022 | Hypothyroidism \|\| id:ebi-a-GCST90029022 | TRUE | reported | igd | 2 | 1 | TRUE | 35.84347664 |
| rs2921053 | C | G | C | G | -0.0065747 | -0.016 | 0.451288 | 0.4991 | FALSE | TRUE | TRUE | finn-b-G6_SLEEPAPNO | 8 | 8319963 | 0.0223 |  | 0.4727 | Sleep apnoea \|\| id:finn-b-G6_SLEEPAPNO | Sleep apnoea | Sleep apnoea \|\| \|\| | TRUE | igd | 8 | 8319963 | 0.00087172 | 3.4002E-14 | 473703 | ebi-a-GCST90029022 | Hypothyroidism \|\| id:ebi-a-GCST90029022 | TRUE | reported | igd | 2 | 1 | FALSE | 56.88523149 |
| rs3087243 | A | G | A | G | -0.0084931 | -0.0091 | 0.450577 | 0.3294 | FALSE | FALSE | FALSE | finn-b-G6_SLEEPAPNO | 2 | 204738919 | 0.0132 |  | 0.490801 | Sleep apnoea \|\| id:finn-b-G6_SLEEPAPNO | Sleep apnoea | Sleep apnoea \|\| \|\| | TRUE | igd | 2 | 204738919 | 0.0004431 | 2.3999E-83 | 473703 | ebi-a-GCST90029022 | Hypothyroidism \|\| id:ebi-a-GCST90029022 | TRUE | reported | igd | 2 | 1 | TRUE | 367.3837486 |
| rs3134996 | T | A | T | A | 0.0122905 | 0.0068 | 0.643252 | 0.5483 | FALSE | TRUE | TRUE | finn-b-G6_SLEEPAPNO | 6 | 32636866 | 0.0135 |  | 0.616401 | Sleep apnoea \|\| id:finn-b-G6_SLEEPAPNO | Sleep apnoea | Sleep apnoea \|\| \|\| | TRUE | igd | 6 | 32636866 | 0.00064986 | 4.2005E-77 | 473703 | ebi-a-GCST90029022 | Hypothyroidism \|\| id:ebi-a-GCST90029022 | TRUE | reported | igd | 2 | 1 | FALSE | 357.6883837 |
| rs3184504 | C | T | C | T | -0.0100364 | 0.0047 | 0.516912 | 0.5912 | FALSE | FALSE | FALSE | finn-b-G6_SLEEPAPNO | 12 | 111884608 | 0.0127 |  | 0.7105 | Sleep apnoea \|\| id:finn-b-G6_SLEEPAPNO | Sleep apnoea | Sleep apnoea \|\| \|\| | TRUE | igd | 12 | 111884608 | 0.00044087 | 6.397E-117 | 473703 | ebi-a-GCST90029022 | Hypothyroidism \|\| id:ebi-a-GCST90029022 | TRUE | reported | igd | 2 | 1 | TRUE | 518.2446518 |
| rs34477738 | G | A | G | A | 0.00427542 | -0.0064 | 0.164489 | 0.2087 | FALSE | FALSE | FALSE | finn-b-G6_SLEEPAPNO | 9 | 5447227 | 0.0155 |  | 0.679599 | Sleep apnoea \|\| id:finn-b-G6_SLEEPAPNO | Sleep apnoea | Sleep apnoea \|\| \|\| | TRUE | igd | 9 | 5447227 | 0.00061887 | 5.3003E-12 | 473703 | ebi-a-GCST90029022 | Hypothyroidism \|\| id:ebi-a-GCST90029022 | TRUE | reported | igd | 2 | 1 | TRUE | 47.72624917 |
| rs34536443 | C | G | C | G | -0.0080139 | -0.0019 | 0.045478 | 0.03039 | FALSE | TRUE | FALSE | finn-b-G6_SLEEPAPNO | 19 | 10463118 | 0.0364 |  | 0.9586 | Sleep apnoea \|\| id:finn-b-G6_SLEEPAPNO | Sleep apnoea | Sleep apnoea \|\| \|\| | TRUE | igd | 19 | 10463118 | 0.00109048 | 2.4998E-13 | 473703 | ebi-a-GCST90029022 | Hypothyroidism \|\| id:ebi-a-GCST90029022 | TRUE | reported | igd | 2 | 1 | TRUE | 54.00756486 |
| rs35074907 | A | G | A | G | 0.00943735 | -0.0732 | 0.019918 | 0.006903 | FALSE | FALSE | FALSE | finn-b-G6_SLEEPAPNO | 19 | 10600418 | 0.0774 |  | 0.3445 | Sleep apnoea \|\| id:finn-b-G6_SLEEPAPNO | Sleep apnoea | Sleep apnoea \|\| \|\| | TRUE | igd | 19 | 10600418 | 0.00157823 | 8E-10 | 473703 | ebi-a-GCST90029022 | Hypothyroidism \|\| id:ebi-a-GCST90029022 | TRUE | reported | igd | 2 | 1 | TRUE | 35.75687323 |
| rs3758212 | T | C | T | C | 0.00258418 | 0.0176 | 0.370126 | 0.3767 | FALSE | FALSE | FALSE | finn-b-G6_SLEEPAPNO | 9 | 127069753 | 0.0128 |  | 0.1694 | Sleep apnoea \|\| id:finn-b-G6_SLEEPAPNO | Sleep apnoea | Sleep apnoea \|\| \|\| | TRUE | igd | 9 | 127069753 | 0.00045723 | 2.1E-08 | 473703 | ebi-a-GCST90029022 | Hypothyroidism \|\| id:ebi-a-GCST90029022 | TRUE | reported | igd | 2 | 1 | TRUE | 31.94358721 |
| rs3775291 | T | C | T | C | -0.0040593 | 0 | 0.297325 | 0.3155 | FALSE | FALSE | FALSE | finn-b-G6_SLEEPAPNO | 4 | 187004074 | 0.0134 |  | 0.9973 | Sleep apnoea \|\| id:finn-b-G6_SLEEPAPNO | Sleep apnoea | Sleep apnoea \|\| \|\| | TRUE | igd | 4 | 187004074 | 0.00048207 | 1.2999E-17 | 473703 | ebi-a-GCST90029022 | Hypothyroidism \|\| id:ebi-a-GCST90029022 | TRUE | reported | igd | 2 | 1 | TRUE | 70.90421672 |
| rs3784099 | A | G | A | G | -0.0031067 | 0.0094 | 0.281314 | 0.3048 | FALSE | FALSE | FALSE | finn-b-G6_SLEEPAPNO | 14 | 68749927 | 0.0136 |  | 0.4876 | Sleep apnoea \|\| id:finn-b-G6_SLEEPAPNO | Sleep apnoea | Sleep apnoea \|\| \|\| | TRUE | igd | 14 | 68749927 | 0.00049082 | 2.7002E-11 | 473703 | ebi-a-GCST90029022 | Hypothyroidism \|\| id:ebi-a-GCST90029022 | TRUE | reported | igd | 2 | 1 | TRUE | 40.06246248 |
| rs3807307 | C | T | C | T | 0.00264478 | -0.0145 | 0.464413 | 0.4286 | FALSE | FALSE | FALSE | finn-b-G6_SLEEPAPNO | 7 | 128579202 | 0.0126 |  | 0.2468 | Sleep apnoea \|\| id:finn-b-G6_SLEEPAPNO | Sleep apnoea | Sleep apnoea \|\| \|\| | TRUE | igd | 7 | 128579202 | 0.00044215 | 1.8E-09 | 473703 | ebi-a-GCST90029022 | Hypothyroidism \|\| id:ebi-a-GCST90029022 | TRUE | reported | igd | 2 | 1 | TRUE | 35.78076881 |
| rs3828887 | T | G | T | G | 0.00533082 | 0.0182 | 0.129005 | 0.138 | FALSE | FALSE | FALSE | finn-b-G6_SLEEPAPNO | 6 | 31440599 | 0.018 |  | 0.312 | Sleep apnoea \|\| id:finn-b-G6_SLEEPAPNO | Sleep apnoea | Sleep apnoea \|\| \|\| | TRUE | igd | 6 | 31440599 | 0.00072476 | 3.5003E-12 | 473703 | ebi-a-GCST90029022 | Hypothyroidism \|\| id:ebi-a-GCST90029022 | TRUE | reported | igd | 2 | 1 | TRUE | 54.10103785 |
| rs3850765 | C | T | C | T | 0.00326522 | -0.0078 | 0.585952 | 0.7061 | FALSE | FALSE | FALSE | finn-b-G6_SLEEPAPNO | 10 | 124139910 | 0.0137 |  | 0.570401 | Sleep apnoea \|\| id:finn-b-G6_SLEEPAPNO | Sleep apnoea | Sleep apnoea \|\| \|\| | TRUE | igd | 10 | 124139910 | 0.00044785 | 6.7004E-14 | 473703 | ebi-a-GCST90029022 | Hypothyroidism \|\| id:ebi-a-GCST90029022 | TRUE | reported | igd | 2 | 1 | TRUE | 53.15738616 |
| rs4293777 | C | G | C | G | 0.00369796 | 0.0137 | 0.472692 | 0.489 | FALSE | TRUE | TRUE | finn-b-G6_SLEEPAPNO | 4 | 10716939 | 0.0125 |  | 0.27 | Sleep apnoea \|\| id:finn-b-G6_SLEEPAPNO | Sleep apnoea | Sleep apnoea \|\| \|\| | TRUE | igd | 4 | 10716939 | 0.00044176 | 7.8001E-17 | 473703 | ebi-a-GCST90029022 | Hypothyroidism \|\| id:ebi-a-GCST90029022 | TRUE | reported | igd | 2 | 1 | FALSE | 70.07251535 |
| rs4409785 | C | T | C | T | 0.0068012 | -0.0197 | 0.172622 | 0.1668 | FALSE | FALSE | FALSE | finn-b-G6_SLEEPAPNO | 11 | 95311422 | 0.0167 |  | 0.2383 | Sleep apnoea \|\| id:finn-b-G6_SLEEPAPNO | Sleep apnoea | Sleep apnoea \|\| \|\| | TRUE | igd | 11 | 95311422 | 0.00058381 | 4.4999E-32 | 473703 | ebi-a-GCST90029022 | Hypothyroidism \|\| id:ebi-a-GCST90029022 | TRUE | reported | igd | 2 | 1 | TRUE | 135.7164516 |
| rs4410767 | C | T | C | T | 0.00525256 | 0.0269 | 0.473325 | 0.4376 | FALSE | FALSE | FALSE | finn-b-G6_SLEEPAPNO | 6 | 32448129 | 0.0153 |  | 0.0787408 | Sleep apnoea \|\| id:finn-b-G6_SLEEPAPNO | Sleep apnoea | Sleep apnoea \|\| \|\| | TRUE | igd | 6 | 32448129 | 0.00069606 | 7.5007E-18 | 473703 | ebi-a-GCST90029022 | Hypothyroidism \|\| id:ebi-a-GCST90029022 | TRUE | reported | igd | 2 | 1 | TRUE | 56.94409429 |
| rs4444866 | T | C | T | C | -0.0033681 | 0.0078 | 0.27653 | 0.2875 | FALSE | FALSE | FALSE | finn-b-G6_SLEEPAPNO | 4 | 40307533 | 0.0138 |  | 0.5714 | Sleep apnoea \|\| id:finn-b-G6_SLEEPAPNO | Sleep apnoea | Sleep apnoea \|\| \|\| | TRUE | igd | 4 | 40307533 | 0.00049634 | 1.1E-11 | 473703 | ebi-a-GCST90029022 | Hypothyroidism \|\| id:ebi-a-GCST90029022 | TRUE | reported | igd | 2 | 1 | TRUE | 46.04751013 |
| rs4687854 | A | G | A | G | 0.00357659 | 0.0049 | 0.194806 | 0.2645 | FALSE | FALSE | FALSE | finn-b-G6_SLEEPAPNO | 3 | 119130373 | 0.0141 |  | 0.7267 | Sleep apnoea \|\| id:finn-b-G6_SLEEPAPNO | Sleep apnoea | Sleep apnoea \|\| \|\| | TRUE | igd | 3 | 119130373 | 0.00055664 | 8.6E-11 | 473703 | ebi-a-GCST90029022 | Hypothyroidism \|\| id:ebi-a-GCST90029022 | TRUE | reported | igd | 2 | 1 | TRUE | 41.28428922 |
| rs4824117 | G | A | G | A | -0.0026495 | -0.0139 | 0.666675 | 0.6947 | FALSE | FALSE | FALSE | finn-b-G6_SLEEPAPNO | 22 | 50895133 | 0.0135 |  | 0.3031 | Sleep apnoea \|\| id:finn-b-G6_SLEEPAPNO | Sleep apnoea | Sleep apnoea \|\| \|\| | TRUE | igd | 22 | 50895133 | 0.00046922 | 2.8E-08 | 473703 | ebi-a-GCST90029022 | Hypothyroidism \|\| id:ebi-a-GCST90029022 | TRUE | reported | igd | 2 | 1 | TRUE | 31.88329014 |
| rs4919342 | C | G | C | G | -0.0027821 | -0.0094 | 0.524435 | 0.5414 | FALSE | TRUE | TRUE | finn-b-G6_SLEEPAPNO | 10 | 101282553 | 0.0125 |  | 0.4495 | Sleep apnoea \|\| id:finn-b-G6_SLEEPAPNO | Sleep apnoea | Sleep apnoea \|\| \|\| | TRUE | igd | 10 | 101282553 | 0.00044134 | 4.5E-10 | 473703 | ebi-a-GCST90029022 | Hypothyroidism \|\| id:ebi-a-GCST90029022 | TRUE | reported | igd | 2 | 1 | FALSE | 39.73831163 |
| rs56249713 | C | T | C | T | -0.0027003 | -0.0208 | 0.419214 | 0.4042 | FALSE | FALSE | FALSE | finn-b-G6_SLEEPAPNO | 18 | 67533332 | 0.0128 |  | 0.1038 | Sleep apnoea \|\| id:finn-b-G6_SLEEPAPNO | Sleep apnoea | Sleep apnoea \|\| \|\| | TRUE | igd | 18 | 67533332 | 0.00044987 | 8.9E-10 | 473703 | ebi-a-GCST90029022 | Hypothyroidism \|\| id:ebi-a-GCST90029022 | TRUE | reported | igd | 2 | 1 | TRUE | 36.02870709 |
| rs56818621 | G | C | G | C | -0.0037024 | 0.0035 | 0.128281 | 0.2568 | FALSE | TRUE | FALSE | finn-b-G6_SLEEPAPNO | 1 | 65435425 | 0.0143 |  | 0.8066 | Sleep apnoea \|\| id:finn-b-G6_SLEEPAPNO | Sleep apnoea | Sleep apnoea \|\| \|\| | TRUE | igd | 1 | 65435425 | 0.00066312 | 5.1E-09 | 473703 | ebi-a-GCST90029022 | Hypothyroidism \|\| id:ebi-a-GCST90029022 | TRUE | reported | igd | 2 | 1 | TRUE | 31.17307084 |
| rs57938373 | T | C | T | C | 0.00408291 | 0.0046 | 0.146978 | 0.1508 | FALSE | FALSE | FALSE | finn-b-G6_SLEEPAPNO | 3 | 39336038 | 0.0174 |  | 0.7887 | Sleep apnoea \|\| id:finn-b-G6_SLEEPAPNO | Sleep apnoea | Sleep apnoea \|\| \|\| | TRUE | igd | 3 | 39336038 | 0.00062245 | 7.5007E-11 | 473703 | ebi-a-GCST90029022 | Hypothyroidism \|\| id:ebi-a-GCST90029022 | TRUE | reported | igd | 2 | 1 | TRUE | 43.02541712 |
| rs58014733 | A | G | A | G | 0.00272327 | 0.0076 | 0.381908 | 0.4021 | FALSE | FALSE | FALSE | finn-b-G6_SLEEPAPNO | 18 | 77184016 | 0.0127 |  | 0.5513 | Sleep apnoea \|\| id:finn-b-G6_SLEEPAPNO | Sleep apnoea | Sleep apnoea \|\| \|\| | TRUE | igd | 18 | 77184016 | 0.0004568 | 1.2E-09 | 473703 | ebi-a-GCST90029022 | Hypothyroidism \|\| id:ebi-a-GCST90029022 | TRUE | reported | igd | 2 | 1 | TRUE | 35.54112042 |
| rs5865 | T | C | T | C | -0.0028646 | -0.0077 | 0.664505 | 0.707 | FALSE | FALSE | FALSE | finn-b-G6_SLEEPAPNO | 2 | 98373006 | 0.0136 |  | 0.5726 | Sleep apnoea \|\| id:finn-b-G6_SLEEPAPNO | Sleep apnoea | Sleep apnoea \|\| \|\| | TRUE | igd | 2 | 98373006 | 0.00046824 | 1.2E-09 | 473703 | ebi-a-GCST90029022 | Hypothyroidism \|\| id:ebi-a-GCST90029022 | TRUE | reported | igd | 2 | 1 | TRUE | 37.42935615 |
| rs60600003 | G | T | G | T | 0.00440281 | 0.0003 | 0.100316 | 0.1018 | FALSE | FALSE | FALSE | finn-b-G6_SLEEPAPNO | 7 | 37382465 | 0.0206 |  | 0.9872 | Sleep apnoea \|\| id:finn-b-G6_SLEEPAPNO | Sleep apnoea | Sleep apnoea \|\| \|\| | TRUE | igd | 7 | 37382465 | 0.00073887 | 9.2999E-10 | 473703 | ebi-a-GCST90029022 | Hypothyroidism \|\| id:ebi-a-GCST90029022 | TRUE | reported | igd | 2 | 1 | TRUE | 35.50790073 |
| rs6111715 | C | G | C | G | -0.003711 | -0.0245 | 0.179515 | 0.1456 | FALSE | TRUE | FALSE | finn-b-G6_SLEEPAPNO | 20 | 17860022 | 0.0177 |  | 0.1677 | Sleep apnoea \|\| id:finn-b-G6_SLEEPAPNO | Sleep apnoea | Sleep apnoea \|\| \|\| | TRUE | igd | 20 | 17860022 | 0.00057551 | 7.8001E-11 | 473703 | ebi-a-GCST90029022 | Hypothyroidism \|\| id:ebi-a-GCST90029022 | TRUE | reported | igd | 2 | 1 | TRUE | 41.57955687 |
| rs61759532 | T | C | T | C | 0.00399086 | -0.0082 | 0.246798 | 0.1898 | FALSE | FALSE | FALSE | finn-b-G6_SLEEPAPNO | 17 | 7240391 | 0.0159 |  | 0.6064 | Sleep apnoea \|\| id:finn-b-G6_SLEEPAPNO | Sleep apnoea | Sleep apnoea \|\| \|\| | TRUE | igd | 17 | 7240391 | 0.00052719 | 1E-14 | 473703 | ebi-a-GCST90029022 | Hypothyroidism \|\| id:ebi-a-GCST90029022 | TRUE | reported | igd | 2 | 1 | TRUE | 57.30515818 |
| rs61776678 | A | G | A | G | -0.0028695 | -0.0112 | 0.410754 | 0.4413 | FALSE | FALSE | FALSE | finn-b-G6_SLEEPAPNO | 1 | 38377021 | 0.0125 |  | 0.3701 | Sleep apnoea \|\| id:finn-b-G6_SLEEPAPNO | Sleep apnoea | Sleep apnoea \|\| \|\| | TRUE | igd | 1 | 38377021 | 0.00044833 | 8.8004E-11 | 473703 | ebi-a-GCST90029022 | Hypothyroidism \|\| id:ebi-a-GCST90029022 | TRUE | reported | igd | 2 | 1 | TRUE | 40.96705801 |
| rs61983206 | T | C | T | C | -0.0036748 | -0.0135 | 0.470275 | 0.4464 | FALSE | FALSE | FALSE | finn-b-G6_SLEEPAPNO | 14 | 106114658 | 0.0132 |  | 0.3044 | Sleep apnoea \|\| id:finn-b-G6_SLEEPAPNO | Sleep apnoea | Sleep apnoea \|\| \|\| | TRUE | igd | 14 | 106114658 | 0.0005022 | 1.5E-13 | 473703 | ebi-a-GCST90029022 | Hypothyroidism \|\| id:ebi-a-GCST90029022 | TRUE | reported | igd | 2 | 1 | TRUE | 53.54487626 |
| rs6456739 | C | T | C | T | -0.0033031 | 0.0117 | 0.559142 | 0.4545 | FALSE | FALSE | FALSE | finn-b-G6_SLEEPAPNO | 6 | 26604140 | 0.0131 |  | 0.3727 | Sleep apnoea \|\| id:finn-b-G6_SLEEPAPNO | Sleep apnoea | Sleep apnoea \|\| \|\| | TRUE | igd | 6 | 26604140 | 0.0005197 | 3E-10 | 473703 | ebi-a-GCST90029022 | Hypothyroidism \|\| id:ebi-a-GCST90029022 | TRUE | reported | igd | 2 | 1 | TRUE | 40.39643353 |
| rs6505765 | G | C | G | C | 0.00340643 | 0.0041 | 0.343863 | 0.3393 | FALSE | TRUE | FALSE | finn-b-G6_SLEEPAPNO | 18 | 12782849 | 0.0131 |  | 0.754899 | Sleep apnoea \|\| id:finn-b-G6_SLEEPAPNO | Sleep apnoea | Sleep apnoea \|\| \|\| | TRUE | igd | 18 | 12782849 | 0.00046501 | 9.7006E-13 | 473703 | ebi-a-GCST90029022 | Hypothyroidism \|\| id:ebi-a-GCST90029022 | TRUE | reported | igd | 2 | 1 | TRUE | 53.66200326 |
| rs66749983 | T | A | T | A | 0.00361082 | -0.0058 | 0.310665 | 0.349 | FALSE | TRUE | FALSE | finn-b-G6_SLEEPAPNO | 13 | 43063831 | 0.0131 |  | 0.6591 | Sleep apnoea \|\| id:finn-b-G6_SLEEPAPNO | Sleep apnoea | Sleep apnoea \|\| \|\| | TRUE | igd | 13 | 43063831 | 0.00047778 | 3.9003E-14 | 473703 | ebi-a-GCST90029022 | Hypothyroidism \|\| id:ebi-a-GCST90029022 | TRUE | reported | igd | 2 | 1 | TRUE | 57.11501465 |
| rs6679677 | A | C | A | C | 0.0203536 | 0.0143 | 0.101076 | 0.1472 | FALSE | FALSE | FALSE | finn-b-G6_SLEEPAPNO | 1 | 114303808 | 0.0175 |  | 0.4125 | Sleep apnoea \|\| id:finn-b-G6_SLEEPAPNO | Sleep apnoea | Sleep apnoea \|\| \|\| | TRUE | igd | 1 | 114303808 | 0.0007301 | 2E-173 | 473703 | ebi-a-GCST90029022 | Hypothyroidism \|\| id:ebi-a-GCST90029022 | TRUE | reported | igd | 2 | 1 | TRUE | 777.1816401 |
| rs6739788 | T | A | T | A | 0.00558581 | -0.0399 | 0.053688 | 0.03627 | FALSE | TRUE | FALSE | finn-b-G6_SLEEPAPNO | 2 | 55862355 | 0.033 |  | 0.2262 | Sleep apnoea \|\| id:finn-b-G6_SLEEPAPNO | Sleep apnoea | Sleep apnoea \|\| \|\| | TRUE | igd | 2 | 55862355 | 0.00097767 | 7.7999E-09 | 473703 | ebi-a-GCST90029022 | Hypothyroidism \|\| id:ebi-a-GCST90029022 | TRUE | reported | igd | 2 | 1 | TRUE | 32.64289205 |
| rs6798068 | A | G | A | G | 0.00282356 | -0.0299 | 0.322403 | 0.2736 | FALSE | FALSE | FALSE | finn-b-G6_SLEEPAPNO | 3 | 108162362 | 0.014 |  | 0.0326002 | Sleep apnoea \|\| id:finn-b-G6_SLEEPAPNO | Sleep apnoea | Sleep apnoea \|\| \|\| | TRUE | igd | 3 | 108162362 | 0.00047155 | 2.2E-09 | 473703 | ebi-a-GCST90029022 | Hypothyroidism \|\| id:ebi-a-GCST90029022 | TRUE | reported | igd | 2 | 1 | TRUE | 35.85453292 |
| rs6833591 | G | A | G | A | -0.0028548 | 0.0016 | 0.347096 | 0.2169 | FALSE | FALSE | FALSE | finn-b-G6_SLEEPAPNO | 4 | 123546282 | 0.0151 |  | 0.917 | Sleep apnoea \|\| id:finn-b-G6_SLEEPAPNO | Sleep apnoea | Sleep apnoea \|\| \|\| | TRUE | igd | 4 | 123546282 | 0.00046426 | 7.5999E-10 | 473703 | ebi-a-GCST90029022 | Hypothyroidism \|\| id:ebi-a-GCST90029022 | TRUE | reported | igd | 2 | 1 | TRUE | 37.81192972 |
| rs6992869 | C | T | C | T | 0.00282023 | 0.0091 | 0.374615 | 0.4284 | FALSE | FALSE | FALSE | finn-b-G6_SLEEPAPNO | 8 | 61395832 | 0.0126 |  | 0.467599 | Sleep apnoea \|\| id:finn-b-G6_SLEEPAPNO | Sleep apnoea | Sleep apnoea \|\| \|\| | TRUE | igd | 8 | 61395832 | 0.00045654 | 3.2E-10 | 473703 | ebi-a-GCST90029022 | Hypothyroidism \|\| id:ebi-a-GCST90029022 | TRUE | reported | igd | 2 | 1 | TRUE | 38.16110133 |
| rs7005834 | T | C | T | C | -0.0032623 | 0.0255 | 0.307465 | 0.2501 | FALSE | FALSE | FALSE | finn-b-G6_SLEEPAPNO | 8 | 134214204 | 0.0144 |  | 0.0753494 | Sleep apnoea \|\| id:finn-b-G6_SLEEPAPNO | Sleep apnoea | Sleep apnoea \|\| \|\| | TRUE | igd | 8 | 134214204 | 0.00047668 | 2.8003E-12 | 473703 | ebi-a-GCST90029022 | Hypothyroidism \|\| id:ebi-a-GCST90029022 | TRUE | reported | igd | 2 | 1 | TRUE | 46.83906587 |
| rs7090530 | A | C | A | C | 0.00402055 | -0.0126 | 0.602441 | 0.6752 | FALSE | FALSE | FALSE | finn-b-G6_SLEEPAPNO | 10 | 6110875 | 0.0133 |  | 0.3412 | Sleep apnoea \|\| id:finn-b-G6_SLEEPAPNO | Sleep apnoea | Sleep apnoea \|\| \|\| | TRUE | igd | 10 | 6110875 | 0.00045087 | 4.7E-19 | 473703 | ebi-a-GCST90029022 | Hypothyroidism \|\| id:ebi-a-GCST90029022 | TRUE | reported | igd | 2 | 1 | TRUE | 79.51745597 |
| rs71508903 | T | C | T | C | 0.00674365 | 0.0199 | 0.194012 | 0.1853 | FALSE | FALSE | FALSE | finn-b-G6_SLEEPAPNO | 10 | 63779871 | 0.0161 |  | 0.2172 | Sleep apnoea \|\| id:finn-b-G6_SLEEPAPNO | Sleep apnoea | Sleep apnoea \|\| \|\| | TRUE | igd | 10 | 63779871 | 0.00056325 | 2.7002E-34 | 473703 | ebi-a-GCST90029022 | Hypothyroidism \|\| id:ebi-a-GCST90029022 | TRUE | reported | igd | 2 | 1 | TRUE | 143.3482085 |
| rs7184802 | A | G | A | G | -0.0035443 | -0.0142 | 0.183616 | 0.1631 | FALSE | FALSE | FALSE | finn-b-G6_SLEEPAPNO | 16 | 50355996 | 0.0168 |  | 0.3971 | Sleep apnoea \|\| id:finn-b-G6_SLEEPAPNO | Sleep apnoea | Sleep apnoea \|\| \|\| | TRUE | igd | 16 | 50355996 | 0.00057027 | 6E-10 | 473703 | ebi-a-GCST90029022 | Hypothyroidism \|\| id:ebi-a-GCST90029022 | TRUE | reported | igd | 2 | 1 | TRUE | 38.62725141 |
| rs72507842 | C | T | C | T | -0.0137469 | 0.0498 | 0.026012 | 0.02341 | FALSE | FALSE | FALSE | finn-b-G6_SLEEPAPNO | 6 | 29765652 | 0.042 |  | 0.2356 | Sleep apnoea \|\| id:finn-b-G6_SLEEPAPNO | Sleep apnoea | Sleep apnoea \|\| \|\| | TRUE | igd | 6 | 29765652 | 0.00185823 | 2.9999E-12 | 473703 | ebi-a-GCST90029022 | Hypothyroidism \|\| id:ebi-a-GCST90029022 | TRUE | reported | igd | 2 | 1 | TRUE | 54.72813044 |
| rs7441808 | G | A | G | A | 0.00391329 | -0.0111 | 0.301062 | 0.2825 | FALSE | FALSE | FALSE | finn-b-G6_SLEEPAPNO | 4 | 26090375 | 0.0138 |  | 0.4206 | Sleep apnoea \|\| id:finn-b-G6_SLEEPAPNO | Sleep apnoea | Sleep apnoea \|\| \|\| | TRUE | igd | 4 | 26090375 | 0.00048064 | 6.4003E-17 | 473703 | ebi-a-GCST90029022 | Hypothyroidism \|\| id:ebi-a-GCST90029022 | TRUE | reported | igd | 2 | 1 | TRUE | 66.28832049 |
| rs75051580 | C | T | C | T | 0.00295585 | 0.0142 | 0.204508 | 0.2542 | FALSE | FALSE | FALSE | finn-b-G6_SLEEPAPNO | 3 | 5012125 | 0.0143 |  | 0.3221 | Sleep apnoea \|\| id:finn-b-G6_SLEEPAPNO | Sleep apnoea | Sleep apnoea \|\| \|\| | TRUE | igd | 3 | 5012125 | 0.00055043 | 4.6E-08 | 473703 | ebi-a-GCST90029022 | Hypothyroidism \|\| id:ebi-a-GCST90029022 | TRUE | reported | igd | 2 | 1 | TRUE | 28.83780283 |
| rs7522243 | G | A | G | A | 0.00246842 | -0.0216 | 0.444211 | 0.3929 | FALSE | FALSE | FALSE | finn-b-G6_SLEEPAPNO | 1 | 110362858 | 0.0127 |  | 0.0896004 | Sleep apnoea \|\| id:finn-b-G6_SLEEPAPNO | Sleep apnoea | Sleep apnoea \|\| \|\| | TRUE | igd | 1 | 110362858 | 0.00044564 | 3E-08 | 473703 | ebi-a-GCST90029022 | Hypothyroidism \|\| id:ebi-a-GCST90029022 | TRUE | reported | igd | 2 | 1 | TRUE | 30.68129456 |
| rs7540342 | C | T | C | T | -0.0029111 | -0.0007 | 0.26365 | 0.2523 | FALSE | FALSE | FALSE | finn-b-G6_SLEEPAPNO | 1 | 38622268 | 0.0143 |  | 0.9631 | Sleep apnoea \|\| id:finn-b-G6_SLEEPAPNO | Sleep apnoea | Sleep apnoea \|\| \|\| | TRUE | igd | 1 | 38622268 | 0.00049978 | 7.3E-09 | 473703 | ebi-a-GCST90029022 | Hypothyroidism \|\| id:ebi-a-GCST90029022 | TRUE | reported | igd | 2 | 1 | TRUE | 33.92726084 |
| rs7582694 | G | C | G | C | -0.0069146 | -0.0111 | 0.774003 | 0.7676 | FALSE | TRUE | FALSE | finn-b-G6_SLEEPAPNO | 2 | 191970120 | 0.0147 |  | 0.4496 | Sleep apnoea \|\| id:finn-b-G6_SLEEPAPNO | Sleep apnoea | Sleep apnoea \|\| \|\| | TRUE | igd | 2 | 191970120 | 0.00052772 | 4.4005E-39 | 473703 | ebi-a-GCST90029022 | Hypothyroidism \|\| id:ebi-a-GCST90029022 | TRUE | reported | igd | 2 | 1 | TRUE | 171.6851505 |
| rs7583027 | C | A | C | A | 0.00290231 | 0.0032 | 0.640988 | 0.6563 | FALSE | FALSE | FALSE | finn-b-G6_SLEEPAPNO | 2 | 62544391 | 0.0131 |  | 0.8101 | Sleep apnoea \|\| id:finn-b-G6_SLEEPAPNO | Sleep apnoea | Sleep apnoea \|\| \|\| | TRUE | igd | 2 | 62544391 | 0.00045991 | 4.6E-10 | 473703 | ebi-a-GCST90029022 | Hypothyroidism \|\| id:ebi-a-GCST90029022 | TRUE | reported | igd | 2 | 1 | TRUE | 39.8245919 |
| rs7596240 | G | A | G | A | 0.00286733 | 0.0084 | 0.270214 | 0.2555 | FALSE | FALSE | FALSE | finn-b-G6_SLEEPAPNO | 2 | 242444173 | 0.0143 |  | 0.5564 | Sleep apnoea \|\| id:finn-b-G6_SLEEPAPNO | Sleep apnoea | Sleep apnoea \|\| \|\| | TRUE | igd | 2 | 242444173 | 0.00049596 | 4.4E-09 | 473703 | ebi-a-GCST90029022 | Hypothyroidism \|\| id:ebi-a-GCST90029022 | TRUE | reported | igd | 2 | 1 | TRUE | 33.42468388 |
| rs761357 | T | A | T | A | 0.00282436 | 0.0041 | 0.375425 | 0.3547 | FALSE | TRUE | FALSE | finn-b-G6_SLEEPAPNO | 6 | 135902599 | 0.0131 |  | 0.7543 | Sleep apnoea \|\| id:finn-b-G6_SLEEPAPNO | Sleep apnoea | Sleep apnoea \|\| \|\| | TRUE | igd | 6 | 135902599 | 0.00045634 | 3.4E-10 | 473703 | ebi-a-GCST90029022 | Hypothyroidism \|\| id:ebi-a-GCST90029022 | TRUE | reported | igd | 2 | 1 | TRUE | 38.30566694 |
| rs7649344 | C | T | C | T | -0.0026115 | 0.0036 | 0.457197 | 0.373 | FALSE | FALSE | FALSE | finn-b-G6_SLEEPAPNO | 3 | 37006396 | 0.0129 |  | 0.778901 | Sleep apnoea \|\| id:finn-b-G6_SLEEPAPNO | Sleep apnoea | Sleep apnoea \|\| \|\| | TRUE | igd | 3 | 37006396 | 0.00044329 | 1.2E-08 | 473703 | ebi-a-GCST90029022 | Hypothyroidism \|\| id:ebi-a-GCST90029022 | TRUE | reported | igd | 2 | 1 | TRUE | 34.7075507 |
| rs76930710 | C | T | C | T | -0.0062507 | -0.0432 | 0.039485 | 0.02573 | FALSE | FALSE | FALSE | finn-b-G6_SLEEPAPNO | 12 | 68434459 | 0.0392 |  | 0.2704 | Sleep apnoea \|\| id:finn-b-G6_SLEEPAPNO | Sleep apnoea | Sleep apnoea \|\| \|\| | TRUE | igd | 12 | 68434459 | 0.00114116 | 2.2E-08 | 473703 | ebi-a-GCST90029022 | Hypothyroidism \|\| id:ebi-a-GCST90029022 | TRUE | reported | igd | 2 | 1 | TRUE | 30.00296881 |
| rs7705526 | A | C | A | C | -0.0028966 | 0.0009 | 0.326372 | 0.3229 | FALSE | FALSE | FALSE | finn-b-G6_SLEEPAPNO | 5 | 1285974 | 0.0133 |  | 0.9438 | Sleep apnoea \|\| id:finn-b-G6_SLEEPAPNO | Sleep apnoea | Sleep apnoea \|\| \|\| | TRUE | igd | 5 | 1285974 | 0.00047674 | 2.2E-09 | 473703 | ebi-a-GCST90029022 | Hypothyroidism \|\| id:ebi-a-GCST90029022 | TRUE | reported | igd | 2 | 1 | TRUE | 36.91608951 |
| rs772920 | G | C | G | C | 0.00363716 | -0.0132 | 0.33577 | 0.3025 | FALSE | TRUE | FALSE | finn-b-G6_SLEEPAPNO | 12 | 56390364 | 0.0135 |  | 0.3297 | Sleep apnoea \|\| id:finn-b-G6_SLEEPAPNO | Sleep apnoea | Sleep apnoea \|\| \|\| | TRUE | igd | 12 | 56390364 | 0.00046672 | 4.1995E-15 | 473703 | ebi-a-GCST90029022 | Hypothyroidism \|\| id:ebi-a-GCST90029022 | TRUE | reported | igd | 2 | 1 | TRUE | 60.73173818 |
| rs7754251 | C | G | C | G | 0.0058572 | 0.0034 | 0.579871 | 0.5508 | FALSE | TRUE | TRUE | finn-b-G6_SLEEPAPNO | 6 | 90989125 | 0.0125 |  | 0.7851 | Sleep apnoea \|\| id:finn-b-G6_SLEEPAPNO | Sleep apnoea | Sleep apnoea \|\| \|\| | TRUE | igd | 6 | 90989125 | 0.00044604 | 4.7E-40 | 473703 | ebi-a-GCST90029022 | Hypothyroidism \|\| id:ebi-a-GCST90029022 | TRUE | reported | igd | 2 | 1 | FALSE | 172.4409007 |
| rs7850258 | G | A | G | A | 0.00996066 | 0.006 | 0.668009 | 0.6535 | FALSE | FALSE | FALSE | finn-b-G6_SLEEPAPNO | 9 | 100549013 | 0.013 |  | 0.6476 | Sleep apnoea \|\| id:finn-b-G6_SLEEPAPNO | Sleep apnoea | Sleep apnoea \|\| \|\| | TRUE | igd | 9 | 100549013 | 0.00046843 | 6.607E-103 | 473703 | ebi-a-GCST90029022 | Hypothyroidism \|\| id:ebi-a-GCST90029022 | TRUE | reported | igd | 2 | 1 | TRUE | 452.1467493 |
| rs7936397 | A | G | A | G | -0.0030228 | 0.0027 | 0.272577 | 0.2038 | FALSE | FALSE | FALSE | finn-b-G6_SLEEPAPNO | 11 | 577534 | 0.0154 |  | 0.8613 | Sleep apnoea \|\| id:finn-b-G6_SLEEPAPNO | Sleep apnoea | Sleep apnoea \|\| \|\| | TRUE | igd | 11 | 577534 | 0.00049599 | 9.1E-10 | 473703 | ebi-a-GCST90029022 | Hypothyroidism \|\| id:ebi-a-GCST90029022 | TRUE | reported | igd | 2 | 1 | TRUE | 37.14334203 |
| rs7937786 | A | C | A | C | -0.0024689 | 0.0028 | 0.391453 | 0.3426 | FALSE | FALSE | FALSE | finn-b-G6_SLEEPAPNO | 11 | 332857 | 0.0132 |  | 0.8311 | Sleep apnoea \|\| id:finn-b-G6_SLEEPAPNO | Sleep apnoea | Sleep apnoea \|\| \|\| | TRUE | igd | 11 | 332857 | 0.00045385 | 3.1E-08 | 473703 | ebi-a-GCST90029022 | Hypothyroidism \|\| id:ebi-a-GCST90029022 | TRUE | reported | igd | 2 | 1 | TRUE | 29.59326438 |
| rs79490353 | C | T | C | T | 0.0106964 | -0.0526 | 0.025322 | 0.01519 | FALSE | FALSE | FALSE | finn-b-G6_SLEEPAPNO | 13 | 28623048 | 0.0519 |  | 0.3111 | Sleep apnoea \|\| id:finn-b-G6_SLEEPAPNO | Sleep apnoea | Sleep apnoea \|\| \|\| | TRUE | igd | 13 | 28623048 | 0.00140319 | 1.3999E-14 | 473703 | ebi-a-GCST90029022 | Hypothyroidism \|\| id:ebi-a-GCST90029022 | TRUE | reported | igd | 2 | 1 | TRUE | 58.10885375 |
| rs8054578 | G | A | G | A | -0.003308 | 0.0097 | 0.774825 | 0.8253 | FALSE | FALSE | FALSE | finn-b-G6_SLEEPAPNO | 16 | 79316815 | 0.0164 |  | 0.5528 | Sleep apnoea \|\| id:finn-b-G6_SLEEPAPNO | Sleep apnoea | Sleep apnoea \|\| \|\| | TRUE | igd | 16 | 79316815 | 0.00052918 | 3.8E-10 | 473703 | ebi-a-GCST90029022 | Hypothyroidism \|\| id:ebi-a-GCST90029022 | TRUE | reported | igd | 2 | 1 | TRUE | 39.07764875 |
| rs9277508 | C | T | C | T | -0.00768 | 0.0037 | 0.305199 | 0.2557 | FALSE | FALSE | FALSE | finn-b-G6_SLEEPAPNO | 6 | 33054177 | 0.0147 |  | 0.7989 | Sleep apnoea \|\| id:finn-b-G6_SLEEPAPNO | Sleep apnoea | Sleep apnoea \|\| \|\| | TRUE | igd | 6 | 33054177 | 0.0005441 | 4.7995E-45 | 473703 | ebi-a-GCST90029022 | Hypothyroidism \|\| id:ebi-a-GCST90029022 | TRUE | reported | igd | 2 | 1 | TRUE | 199.2376331 |
| rs9293291 | A | G | A | G | 0.0029809 | 0.0141 | 0.761967 | 0.7858 | FALSE | FALSE | FALSE | finn-b-G6_SLEEPAPNO | 5 | 71694837 | 0.0152 |  | 0.3519 | Sleep apnoea \|\| id:finn-b-G6_SLEEPAPNO | Sleep apnoea | Sleep apnoea \|\| \|\| | TRUE | igd | 5 | 71694837 | 0.00051995 | 5.1E-09 | 473703 | ebi-a-GCST90029022 | Hypothyroidism \|\| id:ebi-a-GCST90029022 | TRUE | reported | igd | 2 | 1 | TRUE | 32.86749741 |
| rs933243 | A | C | A | C | -0.0056354 | 0.0019 | 0.336133 | 0.3713 | FALSE | FALSE | FALSE | finn-b-G6_SLEEPAPNO | 6 | 167403873 | 0.0129 |  | 0.8853 | Sleep apnoea \|\| id:finn-b-G6_SLEEPAPNO | Sleep apnoea | Sleep apnoea \|\| \|\| | TRUE | igd | 6 | 167403873 | 0.00046624 | 2.6002E-34 | 473703 | ebi-a-GCST90029022 | Hypothyroidism \|\| id:ebi-a-GCST90029022 | TRUE | reported | igd | 2 | 1 | TRUE | 146.0951176 |
| rs9497965 | T | C | T | C | 0.00393518 | -0.0088 | 0.409482 | 0.337 | FALSE | FALSE | FALSE | finn-b-G6_SLEEPAPNO | 6 | 148521292 | 0.0132 |  | 0.5044 | Sleep apnoea \|\| id:finn-b-G6_SLEEPAPNO | Sleep apnoea | Sleep apnoea \|\| \|\| | TRUE | igd | 6 | 148521292 | 0.00044914 | 8.4996E-19 | 473703 | ebi-a-GCST90029022 | Hypothyroidism \|\| id:ebi-a-GCST90029022 | TRUE | reported | igd | 2 | 1 | TRUE | 76.76714774 |
| rs9511151 | A | G | A | G | -0.0062953 | -0.0047 | 0.345979 | 0.3442 | FALSE | FALSE | FALSE | finn-b-G6_SLEEPAPNO | 13 | 24786576 | 0.0131 |  | 0.717399 | Sleep apnoea \|\| id:finn-b-G6_SLEEPAPNO | Sleep apnoea | Sleep apnoea \|\| \|\| | TRUE | igd | 13 | 24786576 | 0.00046541 | 9.8992E-41 | 473703 | ebi-a-GCST90029022 | Hypothyroidism \|\| id:ebi-a-GCST90029022 | TRUE | reported | igd | 2 | 1 | TRUE | 182.962981 |
| rs9521838 | A | G | A | G | -0.0028385 | 0.0286 | 0.233514 | 0.1966 | FALSE | FALSE | FALSE | finn-b-G6_SLEEPAPNO | 13 | 111206226 | 0.0157 |  | 0.0679204 | Sleep apnoea \|\| id:finn-b-G6_SLEEPAPNO | Sleep apnoea | Sleep apnoea \|\| \|\| | TRUE | igd | 13 | 111206226 | 0.00052534 | 4.8E-08 | 473703 | ebi-a-GCST90029022 | Hypothyroidism \|\| id:ebi-a-GCST90029022 | TRUE | reported | igd | 2 | 1 | TRUE | 29.19358505 |
| rs970987 | A | C | A | C | -0.0039158 | 0.0009 | 0.663146 | 0.6576 | FALSE | FALSE | FALSE | finn-b-G6_SLEEPAPNO | 9 | 21585265 | 0.0132 |  | 0.9461 | Sleep apnoea \|\| id:finn-b-G6_SLEEPAPNO | Sleep apnoea | Sleep apnoea \|\| \|\| | TRUE | igd | 9 | 21585265 | 0.00046779 | 3.8001E-17 | 473703 | ebi-a-GCST90029022 | Hypothyroidism \|\| id:ebi-a-GCST90029022 | TRUE | reported | igd | 2 | 1 | TRUE | 70.07065803 |

| Supplementary Table 2 SNPs used as valid instrumental variables for OSA on hypothyroidism. | | | | | | | | | | | | | | | | | | | | | | | | | | | | | | | | | | | | |
| --- | --- | --- | --- | --- | --- | --- | --- | --- | --- | --- | --- | --- | --- | --- | --- | --- | --- | --- | --- | --- | --- | --- | --- | --- | --- | --- | --- | --- | --- | --- | --- | --- | --- | --- | --- | --- |
| SNP | effect_allele.exposure | other_allele.exposure | effect_allele.outcome | other_allele.outcome | beta.exposure | beta.outcome | eaf.exposure | eaf.outcome | remove | palindromic | ambiguous | id.outcome | chr | pos | se.outcome | samplesize.outcome | pval.outcome | outcome | originalname.outcome | outcome.deprecated | mr_keep.outcome | data_source.outcome | pos.exposure | samplesize.exposure | pval.exposure | se.exposure | chr.exposure | id.exposure | exposure | mr_keep.exposure | pval_origin.exposure | data_source.exposure | action | SNP_index | mr_keep | F |
| rs10507084 | T | C | T | C | 0.1085 | -0.0004706 | 0.1793 | 0.059017 | FALSE | FALSE | FALSE | ebi-a-GCST90029022 | 12 | 97753152 | 0.00093691 | 473703 | 0.59 | Hypothyroidism \|\| id:ebi-a-GCST90029022 | Hypothyroidism | Hypothyroidism \|\| \|\| | TRUE | igd | 97753152 |  | 2.7971E-11 | 0.0163 | 12 | finn-b-G6_SLEEPAPNO | Sleep apnoea \|\| id:finn-b-G6_SLEEPAPNO | TRUE | reported | igd | 2 | 1 | TRUE | 44.30821634 |
| rs10928560 | T | C | T | C | -0.0878 | 0.00064618 | 0.1949 | 0.169954 | FALSE | FALSE | FALSE | ebi-a-GCST90029022 | 2 | 136991807 | 0.00059425 | 473703 | 0.31 | Hypothyroidism \|\| id:ebi-a-GCST90029022 | Hypothyroidism | Hypothyroidism \|\| \|\| | TRUE | igd | 136991807 |  | 2.802E-08 | 0.0158 | 2 | finn-b-G6_SLEEPAPNO | Sleep apnoea \|\| id:finn-b-G6_SLEEPAPNO | TRUE | reported | igd | 2 | 1 | TRUE | 30.87982695 |
| rs142006783 | C | T | C | T | 0.1783 | -0.0002442 | 0.03778 | 0.01157 | FALSE | FALSE | FALSE | ebi-a-GCST90029022 | 16 | 74651733 | 0.00215864 | 473703 | 0.88 | Hypothyroidism \|\| id:ebi-a-GCST90029022 | Hypothyroidism | Hypothyroidism \|\| \|\| | TRUE | igd | 74651733 |  | 4.8131E-08 | 0.0327 | 16 | finn-b-G6_SLEEPAPNO | Sleep apnoea \|\| id:finn-b-G6_SLEEPAPNO | TRUE | reported | igd | 2 | 1 | TRUE | 29.73084009 |
| rs4837016 | A | G | A | G | -0.0706 | -0.0010738 | 0.4662 | 0.420941 | FALSE | FALSE | FALSE | ebi-a-GCST90029022 | 9 | 128141809 | 0.00044637 | 473703 | 0.0109999 | Hypothyroidism \|\| id:ebi-a-GCST90029022 | Hypothyroidism | Hypothyroidism \|\| \|\| | TRUE | igd | 128141809 |  | 1.527E-08 | 0.0125 | 9 | finn-b-G6_SLEEPAPNO | Sleep apnoea \|\| id:finn-b-G6_SLEEPAPNO | TRUE | reported | igd | 2 | 1 | TRUE | 31.899904 |
| rs9937053 | A | G | A | G | 0.102 | -0.0001606 | 0.4298 | 0.424188 | FALSE | FALSE | FALSE | ebi-a-GCST90029022 | 16 | 53799507 | 0.00044649 | 473703 | 0.75 | Hypothyroidism \|\| id:ebi-a-GCST90029022 | Hypothyroidism | Hypothyroidism \|\| \|\| | TRUE | igd | 53799507 |  | 4.3192E-16 | 0.0125 | 16 | finn-b-G6_SLEEPAPNO | Sleep apnoea \|\| id:finn-b-G6_SLEEPAPNO | TRUE | reported | igd | 2 | 1 | TRUE | 66.5856 |
